# Metallothionein-2A licenses an S100A8 alarmin program to drive epithelial-mesenchymal transition in pulmonary fibrosis

**DOI:** 10.3389/fimmu.2026.1920411

**Published:** 2026-10-22

**Authors:** Rui Fan, Ying Liu, Guodong Zang, Daowei Li

**Affiliations:** 1 Department of Respiratory and Critical Care Medicine, Shandong Provincial Hospital, Jinan, Shandong, China; 2 Department of Pulmonary Medicine, Xiangyang Hospital Affiliated to Hubei University of Chinese Medicine, Xiangyang, Hubei, China; 3 Hubei Provincial Clinical Research Center for Integrative Medicine in Cancer Green Therapy, Xiangyang, Hubei, China; 4 Department of Respiratory and Critical Care Medicine, Shandong University of Traditional Chinese Medicine Affiliated Hospital, Jinan, Shandong, China

**Keywords:** epithelial-mesenchymal transition, interpretable machine learning, MT2A, pulmonary fibrosis, S100A8, virtual gene knockout

## Abstract

**Background:**

Pulmonary fibrosis (PF) is a progressive interstitial lung disease driven in part by epithelial–mesenchymal transition (EMT), but the upstream epithelial regulators remain undefined, and conventional screens rarely separate causal drivers from bystanders.

**Methods:**

We integrated interpretable machine learning (SHAP-interpreted LASSO across five transcriptomic cohorts), single-cell virtual gene knockout (VGKO), protein–protein docking with molecular dynamics, co-immunoprecipitation and surface plasmon resonance, a bleomycin mouse model, and loss-/gain-of-function studies in TGF-β1-stimulated BEAS-2B cells, complemented by AI-guided drug screening.

**Results:**

SHAP nominated *MT2A* as a positively weighted predictor broadly expressed across multiple cell subpopulations, and in silico knockout nominated *S100A8* as its leading candidate downstream target, yielding a directional *MT2A*→*S100A8* hypothesis. Docking and molecular dynamics predicted a stable *MT2A*–*S100A8* complex, co-immunoprecipitation recovered both proteins from the same complex in cells, and surface plasmon resonance measured direct binding with an apparent dissociation constant of 88 ± 20 nM. *MT2A* and *S100A8* were co-upregulated in fibrotic mouse lung. In epithelial cells, *MT2A* knockdown reduced *S100A8* and reversed the EMT signature, whereas overexpression amplified it. Reciprocal epistasis placed *S100A8* downstream of *MT2A* as its obligate effector: re-expressing *S100A8* restored EMT in *MT2A*-depleted cells, and silencing *S100A8* abolished the pro-fibrotic effect of *MT2A* overexpression. AI screening nominated BRD-K54403101 as a candidate *MT2A* binder.

**Conclusion:**

We identify *MT2A* as broadly expressed across single-cell clusters and show that in bronchial epithelial cells *MT2A* sustains *S100A8*, which is necessary and sufficient to drive the EMT program. The experimentally defined *MT2A-S100A8* axis is a tractable target in pulmonary fibrosis.

## 1 Introduction

Pulmonary fibrosis (PF), of which idiopathic pulmonary fibrosis (IPF) is the most aggressive form, is a chronic and progressive interstitial lung disease characterized by relentless scarring of the lung parenchyma, declining gas exchange and a median survival of three to five years after diagnosis (1, 2). Approved antifibrotic agents—pirfenidone (3, 4), nintedanib (5, 6), and the preferential phosphodiesterase-4B (PDE4B) inhibitor nerandomilast (7, 8)—modestly slow lung-function decline but neither halt nor reverse established fibrosis, and are frequently limited by toxicity and high discontinuation rates. Closing this therapeutic gap requires identifying the upstream regulators that convert a reparative response into pathological remodeling.

The alveolar and airway epithelium is increasingly recognized as an initiating compartment in this process. Repetitive micro-injury provokes aberrant epithelial activation, impaired regeneration and acquisition of a pro-fibrotic secretory phenotype, in part through epithelial–mesenchymal transition (EMT), during which epithelial cells lose apical–basal polarity and cell–cell adhesion, downregulate E-cadherin (*CDH1*, E-cad), and gain mesenchymal markers including vimentin (VIM), fibronectin 1 (FN1) and type I collagen (COL1A1) (9). Identifying the molecular switches that initiate this epithelial program is therefore central to understanding fibrotic progression.

A major obstacle is that conventional differential-expression screens rank genes by association but often cannot distinguish drivers from bystanders, nor specify the direction of causal influence. Recent computational advances address this limitation directly: interpretable machine learning quantifies and signs each feature’s contribution to a phenotype, while single-cell virtual gene knockout perturbs an inferred gene-regulatory network in silico to predict the transcriptional consequences of removing a candidate driver. Coupling these complementary approaches offers a principled route from correlation to a testable, direction-aware causal hypothesis.

Applying this strategy to PF, our pipeline converged on two effectors that recur in fibrotic and inflammatory tissue but have not been mechanistically linked. Metallothionein-2A (MT2A) is a small, cysteine-rich, metal-binding protein implicated in zinc homeostasis, redox buffering and the modulation of inflammatory signaling, yet its role in lung fibrosis remains essentially uncharacterized (10, 11). S100A8, a calcium-binding alarmin, is a potent amplifier of innate-immune and chemotactic signaling and a recognized marker of inflammatory tissue states (12–14). We therefore hypothesized that *MT2A* acts upstream to sustain *S100A8* expression, and that this directional *MT2A-S100A8* axis drives epithelial EMT and thereby promotes fibrotic progression in PF.

To test this hypothesis, interpretable machine learning across multiple cohorts nominated *MT2A* as a high-priority, positively weighted predictor; Single-cell profiling showed its robust expression across major immune lineages, and virtual knockout nominated *S100A8* as its dominant downstream target. Given that *MT2A* is a broadly induced stress-response protein and *S100A8* is induced in both myeloid and epithelial compartments during fibrosis, we tested the predicted MT2A-S100A8 axis in bronchial epithelial cells, where EMT originates. We then tested the predicted *MT2A-S100A8* interaction structurally and biochemically, including by surface plasmon resonance, defined the hierarchy of the axis through loss-of-function, gain-of-function and reciprocal epistasis experiments in epithelial cells, confirmed co-regulation *in vivo*, and used AI-guided screening to prioritize a candidate *MT2A*-targeting small molecule. Together, these results define an *MT2A-S100A8* axis driving epithelial EMT in PF.

## 2 Materials and methods

### 2.1 Data acquisition and preprocessing

Transcriptomic datasets were obtained from the Gene Expression Omnibus (GEO) database (15, 16). Raw data in MINiML format underwent a standardized preprocessing pipeline. Non-normalized data were taken log_2_ transformation, and quantile normalization was applied to ensure comparability across samples. Probe IDs were mapped to official gene symbols using the corresponding platform annotation files; probes matching multiple genes were excluded, and expression values for genes matched by multiple probes were averaged (17). To address technical variability, batch effects were mitigated using the removeBatchEffect function from the limma R package. Data quality and the effectiveness of normalization and batch correction were assessed visually using Principal Component Analysis (PCA) plots.

### 2.2 Differential expression and co-expression network analysis

Differential expression analysis between experimental groups was performed using the limma package in R (v 4.5.2). Statistically significant differentially expressed genes (DEGs) were defined as those with an adjusted P < 0.05 (Benjamini-Hochberg correction) and an absolute log_2_ |FC| > 0.585. To identify gene modules highly correlated with the disease phenotype, a weighted gene co-expression network analysis (WGCNA) was constructed (18). The soft-thresholding power was selected to ensure a scale-free topology. Genes were clustered into modules based on a topological overlap matrix (TOM) (19). The module-trait relationships were calculated, and the module (MEblack) showing the highest positive correlation with the PF phenotype was selected for downstream analysis.

### 2.3 Functional enrichment and protein-protein interaction analysis

The genes from the principal WGCNA module and the DEGs list were intersected to derive a strong collection of PF-related genes. Functional enrichment analysis, encompassing Gene Ontology (GO) and Kyoto Encyclopedia of Genes and Genomes (KEGG) pathway analyses, was conducted on this gene collection utilizing the clusterProfiler R package. A protein-protein interaction (PPI) network was established utilizing the STRING database (https://string-db.org/) (20) and visualized in cytoscape to discern hub nodes according to network Degree.

### 2.4 Single-cell RNA sequencing analysis

Single-cell RNA sequencing (scRNA-seq) data from peripheral blood mononuclear cells (PBMCs) in GSE233844 were analyzed, comprising six healthy controls, six patients with stable IPF and six with progressive IPF samples (the latter two groups collectively treated as the PF group) (21). Data were processed using the Seurat R package (22, 23). Cells with excessive mitochondrial gene expression or outlying feature counts were removed. After normalization, highly variable genes were selected for PCA, followed by graph-based clustering and t-SNE visualization. Ten major PBMC populations were annotated using canonical marker genes, and cluster-specific markers were identified.

### 2.5 Machine learning and SHapley Additive exPlanations analysis

The intersection of genes from DEG and WGCNA analyses was used as input features. 112 ML prediction models were constructed based on transcriptome data, and their performance was validated through independent cohorts using Receiver Operating Characteristic curve (ROC) (24). The model exhibiting the highest mean Area Under the Curve (AUC) was selected as the optimal predictor. SHapley Additive exPlanations (SHAP) analysis was subsequently applied to this best-performing model (LASSO) to quantify both the magnitude and direction of each selected gene’s contribution to the prediction (25). The expression of the final hub genes was validated in an independent clinical cohort, and their diagnostic performance was assessed by ROC analysis (26–28). To enable an objective, hypothesis-driven selection of a single gene for downstream mechanistic study, candidate-prioritization criteria were defined *a priori*: (i) a positive SHAP contribution direction in which higher expression raises the model’s predicted probability of fibrosis; (ii) broad, robust expression across major immune lineages nominated by single-cell analysis; (iii) concordance between the SHAP-inferred direction and the differential-expression direction in PF; and (iv) the absence of a previously defined mechanistic role in pulmonary fibrosis.

### 2.6 Virtual gene knockout and AI-driven drug screening

Causal drivers were inferred by virtual gene knockout (VGKO) from single-cell data using scTenifoldKnk, which constructs a tensor-decomposition–based gene-regulatory network and quantifies the transcriptome-wide consequences of in silico deletion of a target gene (29, 30). Knockout of the prioritized gene (*MT2A*) was performed, and differentially perturbed genes were ranked to nominate downstream effectors and analyzed by gene-set enrichment to identify affected pathways (31). Top-ranking compounds were computationally validated through molecular docking against experimental or AlphaFold2-predicted structures, AI-predicted ADMET profiling, and explainable AI (XAI) techniques to interpret the chemical and structural basis of the predictions (31, 32). Candidate compounds were then prioritized against the resulting perturbation signature using DrugReflector, which scores molecules by structural similarity to the target binding pocket or by phenotypic similarity to a beneficial transcriptional signature.

### 2.7 Protein-protein docking and MD simulation

The MT2A-S100A8 protein–protein complex was modeled by molecular docking using experimentally determined or homology-modeled structures as initial conformations. The resulting complex was solvated explicitly in a TIP3P water box, with protein chains parameterized using the AMBER ff14SB force field (33). System charge was neutralized and ionic strength adjusted by addition of Na^+^/Cl⁻ counterions. All MD simulations were performed using GROMACS 2022 (34). The system underwent energy minimization followed by sequential NVT and NPT equilibration, then production MD simulation. Trajectory convergence was assessed by monitoring RMSD, radius of gyration (Rg), and RMSF; solvent-accessible surface area (SASA) and hydrogen-bond occupancy were used to characterize interface stability. Binding free energy was estimated by MM-PBSA over equilibrated trajectory frames, with per-residue decomposition to identify energetically dominant interface hotspots (35).

### 2.8 Ligand-protein docking and MD simulation

Molecular docking of BRD-K54403101 against MT2A was performed to generate the initial protein–ligand complex, with ligand parameters derived from GAFF2 and AM1-BCC partial charges. The solvated system was prepared identically to the protein–protein system above (AMBER ff14SB/TIP3P; Na^+^/Cl⁻ neutralization) (33), and subjected to the same three-stage equilibration protocol prior to production MD using GROMACS 2022 (34). Trajectory stability was evaluated via RMSD, Rg, RMSF, SASA, and hydrogen-bond persistence. MM-PBSA binding free energy calculations were carried out on representative snapshots, decomposed into van der Waals, electrostatic, polar solvation (PB), and nonpolar solvation (SA) components (35). Free energy landscapes were constructed from two-dimensional probability distributions of RMSD and Rg to characterize dominant conformational states.

### 2.9 Co-immunoprecipitation

HEK293T cells (Procell, CL-0005) were co-transfected with pcDNA3.1-Flag-S100A8 and pcDNA3.1-HA-MT2A using Lipofectamine 3000 (Thermo Fisher Scientific, L3000015). After 48 h, cells were lysed in IP lysis buffer (50 mM Tris-HCl pH 7.4, 150 mM NaCl, 1% NP-40, 5% glycerol) with protease and phosphatase inhibitors, omitting chelating agents to preserve the zinc-loaded conformation of MT2A. Cleared lysates (10% retained as input) were incubated overnight at 4 °C with anti-Flag (Thermo Fisher Scientific, MA1-91878), anti-HA (Proteintech, 51064-2-AP) or species-matched normal IgG, followed by Protein A/G magnetic beads for 4 h. Washed immune complexes and inputs were resolved by Tricine–SDS-PAGE, transferred to glutaraldehyde-fixed 0.2-µm PVDF membranes to retain the 7.3-kDa HA-MT2A, and probed in parallel with anti-Flag and anti-HA alongside molecular-mass markers, so that both partners were detected in every immunoprecipitation.

### 2.10 Surface plasmon resonance

Measurements were performed on a Biacore T200 instrument (Cytiva) at 25 °C. A CM5 sensor chip was activated with 0.4 M EDC and 0.1 M NHS (1:1, 5 µl min⁻¹, 20 min), and recombinant human MT2A (55 µg ml⁻¹ in 10 mM sodium acetate, pH 5.0) was immobilized on flow cell 2 to ~5,000 RU; flow cell 1 was activated and blocked without protein as a reference surface. Both surfaces were blocked with 1 M ethanolamine-HCl, pH 8.5. Recombinant human S100A8 was diluted in running buffer (PBS, 0.05% Tween-20) to 7.5, 15, 30, 62.5, 125 and 250 nM and injected over both flow cells at 30 µl min⁻¹ with 180 s association and 300 s dissociation; buffer alone was injected as a blank and surfaces were regenerated with 10 mM NaOH between cycles. Sensorgrams were double-referenced against the reference flow cell and the buffer blank. Because MT2A folding and S100A8 function are metal-dependent and neither protein was supplemented with metal beyond its supplied formulation, the fitted KD should be regarded as an estimate for the proteins as prepared. Because the interaction approached but did not reach a plateau within the injection, responses averaged over the last 10 s of injection were fitted to a 1:1 steady-state affinity model, R = Rmax·C/(KD + C) + offset, in Biacore Evaluation Software, and the fit was compared with a non-saturable linear model by residual sum of squares. A single orientation was tested (MT2A immobilized, S100A8 in solution). Each concentration series was measured once and the reported values are from one representative series.

### 2.11 Animal models and BLM treatment

Specific pathogen-free (SPF) C57BL/6 mice (male, 6 weeks old) were obtained from Beijing Vital River Laboratory Animal Technology Co., Ltd. (Beijing, China). Mice were randomly assigned to the control or BLM group using a random-number table (n = 6 per group). Sample size was based on our preliminary experiments and previous studies of the BLM model; no animals were excluded from the analysis. For induction of the PF model, mice were anesthetized by intraperitoneal injection of freshly prepared 2,2,2-tribromoethanol (Avertin; 250 mg/kg body weight) and received a single intratracheal instillation of BLM (3 mg/kg body weight, MedChemExpress) dissolved in sterile saline. Mice in the control group received an equal volume of sterile saline. At 21 days post-instillation, mice were deeply anesthetized by intraperitoneal injection of 2,2,2-tribromoethanol (Avertin; 250 mg/kg body weight) and euthanized by exsanguination via cardiac puncture, after which lung tissues and serum were rapidly harvested for subsequent histological and biochemical analyses. All anesthesia and euthanasia procedures were performed in accordance with the American Veterinary Medical Association (AVMA) Guidelines for the Euthanasia of Animals (2020). All animal experiments were approved by the Institutional Animal Care and Use Committee (IACUC) of Shandong Provincial Hospital (No. 2024-038) and conducted in accordance with relevant institutional and national guidelines and regulations.

### 2.12 Histology and fibrosis assessment

Harvested lung tissues were fixed in 4% paraformaldehyde for 24 hours, embedded in paraffin, and sectioned at 5 μm. Sections were stained with hematoxylin and eosin (H&E) to assess general morphology and inflammatory cell infiltration. To evaluate collagen deposition, sections were stained with Masson’s trichrome and Sirius Red using standard protocols. The severity of PF was quantitatively evaluated in a blinded manner utilizing the Alveolar septal thickness (AST). The collagen volume fraction (CVF) was determined by calculating the ratio of the Masson- or Sirius Red-positive fibrotic area to the total tissue area using Image J software.

### 2.13 Immunohistochemistry

Paraffin-embedded lung sections were deparaffinized, rehydrated, and subjected to heat-induced antigen retrieval in sodium citrate buffer. Subsequent immunohistochemistry (IHC) procedures included quenching endogenous peroxidase with 3% H_2_O_2_ and blocking with 5% BSA; sections were incubated overnight at 4°C with anti-MT2A (1:200) and anti-S100A8 (1:200) antibodies. HRP-conjugated secondary antibody was applied for 1 h at room temperature, followed by DAB visualization and hematoxylin counterstaining. Average optical density of positive staining in IHC images was quantified using ImageJ software.

### 2.14 Enzyme-linked immunosorbent assay

Serum levels of TNF-α, IL-6, IL-1β, CXCL1, and CCL3 were measured using commercially available ELISA kits (TNF-α, 88-7346-88; IL-6, 88-7064-88; IL-1β, 88-7013A-88; CXCL1, EMCXCL1; CCL3, 88-7035-88; all from Elabscience, Wuhan, China) according to the manufacturer’s instructions, with absorbance read at 450 nm and cytokine concentrations calculated from standard curves by four-parameter logistic (4-PL) regression.

### 2.15 Cell culture and transfection

Human bronchial epithelial BEAS-2B cells (ATCC, CRL-9609; authenticated by STR profiling, mycoplasma-free) were cultured in RPMI-1640 medium supplemented with 10% fetal bovine serum and 1% penicillin/streptomycin at 37°C in a humidified atmosphere containing 5% CO_2_. For loss-of-function studies, cells were transfected with *MT2A*-targeting small interfering RNA (si*MT2A*; 50 nM), S100A8-targeting siRNA (siS100A8; 50 nM) or a non-targeting scrambled negative control siRNA (siNC) using Lipofectamine 3000 transfection reagent (Thermo Fisher Scientific, L3000015) in Opti-MEM reduced-serum medium, following the manufacturer’s protocol; knockdown efficiency was confirmed at both mRNA and protein levels at 48 h post-transfection. For gain-of-function studies, cells were transiently transfected with pcDNA3.1-based *MT2A* (OE-*MT2A*) or S100A8 (OE-S100A8) overexpression constructs, or the corresponding empty vector, using the same transfection system, and overexpression was validated 48 h after transfection prior to downstream functional assays. Transfection efficiency was confirmed at both mRNA and protein levels prior to downstream functional assays. Cells were co-transfected with siRNA (50 nM) and plasmid (1 ug per well of a six-well plate), each pre-complexed with Lipofectamine 3000 and combined at 1:1; 24 h later the medium was replaced and TGF-β1 (15 ng/mL) was added, and cells were harvested 48 h after stimulation.

### 2.16 Cell viability assay

Cell viability was assessed using a Cell Counting Kit-8 (CCK-8; Dojindo, Kumamoto, Japan). Cells were seeded into 96-well plates at 5 × 10³ cells per well in 100 µl complete medium and allowed to adhere overnight. After the indicated treatments, 10 µl CCK-8 reagent was added to each well and plates were incubated at 37 °C for 2 h in the dark. Absorbance at 450 nm was recorded on a microplate reader (Synergy H1, BioTek, USA), with reference absorbance at 650 nm. Wells containing medium and CCK-8 without cells served as blanks, and viability was expressed relative to untreated controls. Each condition was assayed in five technical replicates across three independent experiments.

### 2.17 Flow cytometry

Apoptosis was quantified by flow cytometry using an Annexin V–FITC/propidium iodide (PI) apoptosis detection kit (BMS500FI-300, Invitrogen/Thermo Fisher Scientific). Following treatment, cells were harvested by trypsinization without EDTA, washed twice in cold PBS and resuspended in 1× binding buffer at 1 × 10^6^ cells ml⁻¹. Aliquots of 100 µl were stained with 5 µl Annexin V–FITC and 5 µl PI for 15 min at room temperature in the dark, after which 400 µl binding buffer was added. Samples were analyzed within 1 h on a flow cytometer (BD FACSCanto II, BD Biosciences), collecting at least 10,000 events per sample. Debris and doublets were excluded by forward- and side-scatter gating, and viable (Annexin V⁻/PI⁻), early-apoptotic (Annexin V^+^/PI⁻) and late-apoptotic (Annexin V^+^/PI^+^) populations were quantified with FlowJo software (v10.8, BD Biosciences). Compensation was performed using single-stained controls.

### 2.18 Immunofluorescence staining

Cells were fixed in 4% paraformaldehyde for 15 min, permeabilized with 0.1% Triton X-100 for 10 min, and blocked with 5% BSA for 1 h at room temperature. The cells were incubated overnight at 4 °C with rabbit anti-S100A8 (1:200; Proteintech, 15792-1-AP) and mouse anti-MT2A (1:200; Sigma-Aldrich, SAB1402848, clone 6G2) primary antibodies, followed by species-appropriate Alexa Fluor–conjugated goat anti-rabbit and goat anti-mouse secondary antibodies for 1 h in the dark. Nuclei were counterstained with DAPI. Images were captured under a fluorescence microscope, and co-localization of MT2A and S100A8 was quantified using ImageJ; Pearson’s correlation coefficient was calculated for individual cells with automatic Costes thresholding.

### 2.19 Migration, invasion, and proliferation assays

Cell migration and invasion were assessed using Transwell chambers (8 μm pore size; Corning). For migration assays, cells were seeded into the upper chamber in serum-free medium, with complete medium placed in the lower chamber as a chemoattractant. For invasion assays, the upper chamber membrane was pre-coated with Matrigel (BD Biosciences) prior to cell seeding. Following 24 h of incubation, non-migrated or non-invaded cells on the upper surface were removed, and cells on the lower membrane surface were fixed with 4% paraformaldehyde, stained with crystal violet, and counted in five randomly selected fields per well under a light microscope. Cell proliferation was evaluated using the EdU incorporation assay (EdU Cell Proliferation Kit; servicebio) according to the manufacturer’s protocol. Briefly, cells were incubated with EdU (10 μM) for 2 h, fixed, permeabilized, and subjected to click chemistry reaction, followed by nuclear counterstaining with DAPI. EdU-positive cells were imaged and quantified under a fluorescence microscope in at least five randomly selected fields.

### 2.20 Quantitative real-time PCR

Total RNA was extracted from BEAS-2B cells using TRIzol reagent (Invitrogen) and reverse-transcribed into cDNA using a reverse transcription kit (Takara) according to the manufacturer’s instructions. Quantitative real-time PCR (qRT-PCR) was performed on a CFX96 real-time PCR system (Bio-Rad) using SYBR Green Master Mix, with GAPDH serving as the internal reference gene. Relative mRNA expression levels were calculated using the 2^−ΔΔCt^ method. The primer sequences used were as follows: GAPDH forward 5’-GGAGCGAGATCCCTCCAAAAT-3’, reverse 5’-GGCTGTTGTCATACTTCTCATGG-3’; *MT2A* forward 5’-CGTGGGCTGTGCGAAGTGCTC-3’, reverse 5’-AAAGGCTAGGCTTCTACATGGTC-3’; *S100A8* forward 5’-ATGCCGTCTACAGGGATGAC-3’, reverse 5’-ACGCCCATCTTTATCACCAG-3’; *COL1A1* forward 5’-GATTCCCTGGACCTAAAGGTGC-3’, reverse 5’-AGCCTCTCCATCTTTGCCAGCA-3’; *FN1* forward 5’-ACAACACCGAGGTGACTGAGAC-3’, reverse 5’-GGACACAACGATGCTTCCTGAG-3’; *VIM* forward 5’-CTTGAACGGAAAGTGGAATCCT-3’, reverse 5’-GTCAGGCTTGGAAACGTCC-3’; *CDH1* forward 5’-AACTGCATGAAGGCGGGAAT-3’, reverse 5’-TTTCGAGTCACTTCCGGTCG-3’.

### 2.21 Western blotting

Cells were lysed in RIPA buffer supplemented with protease and phosphatase inhibitors, and protein concentrations were determined using a BCA protein assay kit (Thermo Fisher Scientific, A55865). Equal amounts of protein were separated by SDS-PAGE and transferred onto PVDF membranes, which were subsequently blocked with 5% non-fat milk for 1 h at room temperature and incubated with primary antibodies against MT2A (1:1000, Proteintech, 20809-1-AP), S100A8 (1:1000, Proteintech, 15792-1-AP), COL1A1 (1:1000, Proteintech, 67288-1-Ig), VIM (1:5000, Proteintech, 10366-1-AP), FN1 (1:1000, Proteintech, 15613-1-AP), E-cadherin (1:10000, Proteintech, 20874-1-AP), and GAPDH (1:50000, Proteintech, 10494-1-AP) overnight at 4 °C, followed by incubation with HRP-conjugated goat anti-rabbit/anti-mouse IgG secondary antibodies (1:10000, Proteintech, SA00001-2/SA00001-1) for 1 h at room temperature. Protein bands were visualized using an enhanced chemiluminescence (ECL) detection system and quantified by densitometric analysis using Image J software.

### 2.22 Statistical analysis

All *in vitro* experiments at least three independent biological replicates. Technical wells and imaging fields were averaged within each biological replicate. Data are mean ± SD, with individual replicates shown. Analyses used GraphPad Prism v10.6.0. Normality and variance homogeneity were assessed before parametric testing. Two-group comparisons used unpaired two-tailed Student’s t-tests, with Welch’s correction when variances differed. Three or more groups were compared by one-way ANOVA with Tukey’s correction. Exact comparisons and significance symbols are defined in figure legends. P < 0.05 (two-tailed) was significant. Animal n = 6 per group was based on preliminary experiments and previous bleomycin studies; no formal power calculation was performed.

## 3 Results

### 3.1 DEGs analysis based on GEO database

This study obtained five separate gene expression datasets from the GEO database (GSE17978, GSE32537, GSE40839, GSE53845 and GSE47460) to uncover hub genes linked to PF pathogenesis. The first four datasets (GSE17978, GSE32537, GSE40839, and GSE53845) were pooled to constitute the training set for initial gene screening, whereas GSE47460 was reserved as an validation set (**Table 1**).

**Table 1:** GEO data.

GEO accession	Platforms	Control samples	Experimental samples	Subject type	References
GSE17978	GPL570	20	38	IPF	(36)
GSE32537	GPL6244	50	167	IPF/NSIP/RB-ILD/DIP/UF	(37)
GSE40839	GPL96	10	11	scleroderma-ILD/UIP	(38)
GSE53845	GPL6480	8	40	IPF	(39)
GSE47460A	GPL6480	17	61	ILD	(40)
GSE47460B	GPL14550	91	193	ILD	

Batch correction was applied to minimize technical variation introduced across different experimental runs, thereby improving the accuracy of downstream analyses in capturing biologically meaningful differences. Initially, we normalized and corrected for batch effects in the training set to eliminate non-biological variation caused by differences in experimental platforms and techniques (**Figures 1A, B**). Subsequently, differential expression analysis was performed on the corrected data using R. A total of 406 DEGs (191 upregulated while 215 downregulated) were identified, following stringent statistical criteria. Differential expression results were visualized via a volcano plot (**Figure 1C**) and a hierarchical clustering heatmap (**Figure 1D**). The heat map visually demonstrates the expression patterns of the 30 most significant DEGs across each sample while the volcano plot illustrates the differences in gene expression profiles between the PF and Control groups. In the PF group, *COMP, ENC1*, and *MFAP2* were consistently upregulated, whereas *CSF3R, IL18RAP, and MGAM* were significantly downregulated. It is also worth noting that *MT2A* is highly expressed in the PF group.

**Figure 1:**
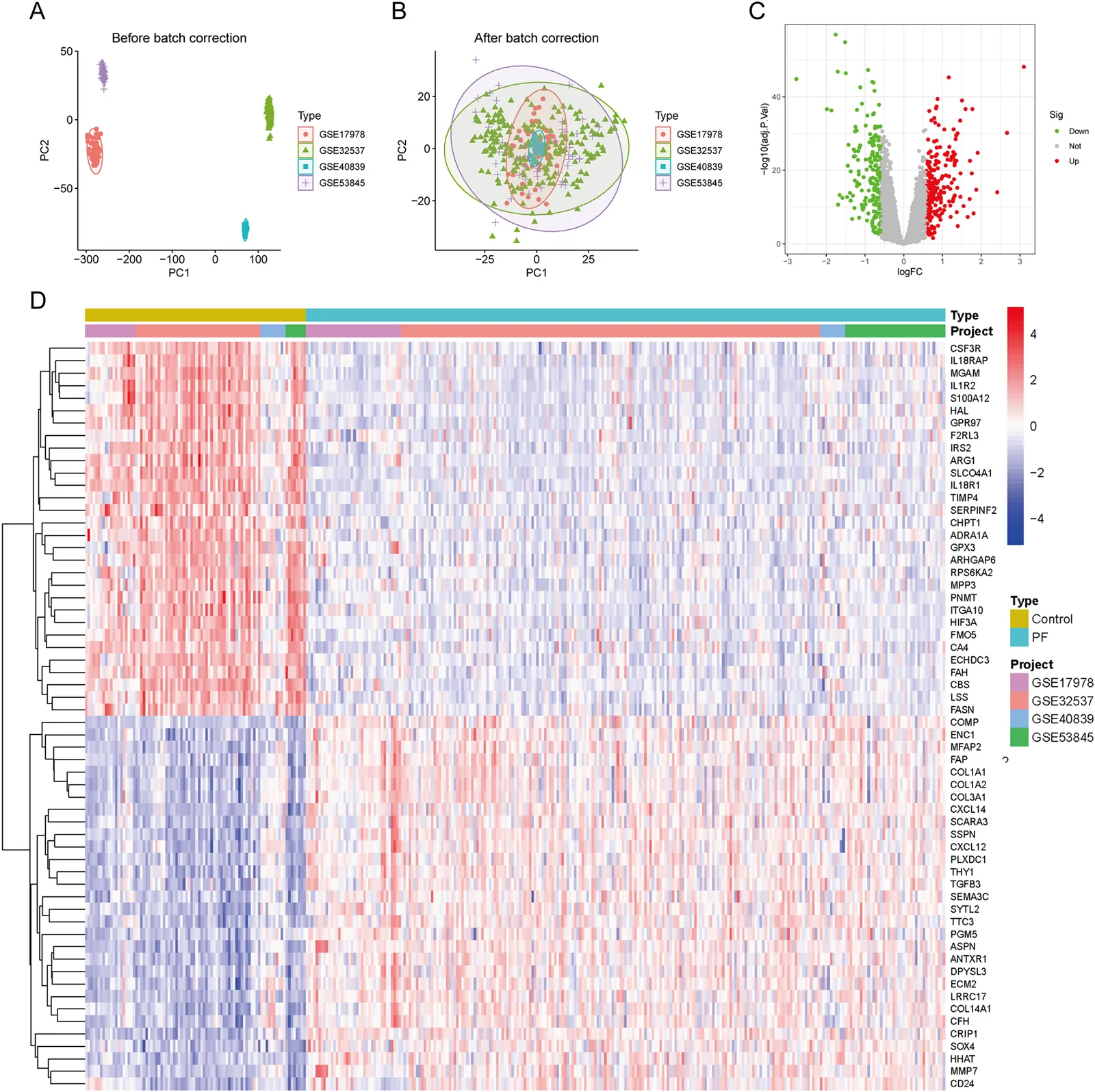
DEGs Analysis of PF. **(A, B)** Comparison of PCA plots before **(A)** and after **(B)** batch correction demonstrates the effective mitigation of batch-specific clustering. **(C)** Volcano plot of DEGs. Genes are plotted based on the magnitude of log_2_ |FC| and statistical p value. Red dots represent significantly upregulated genes, green dots represent significantly downregulated genes, and gray dots indicate non-significant genes. **(D)** Heatmap displays gene expression levels, with red and blue representing high and low expression relative to the mean, respectively. Rows represent individual genes, while columns represent sample origins.

### 3.2 WGCNA analysis and identification of PF-associated genes

We applied WGCNA to identify gene co-expression modules associated with the PF phenotype. A scale-free network was constructed and genes were assigned to distinct color-coded modules. Module–trait correlation analysis identified the black module (MEblack) as the most strongly disease-associated module, showing the highest negative correlation with control status and a correspondingly strong positive correlation with PF (|cor| = 0.70, p = 3 × 10⁻^5^²) (**Figures 2A, B, D**). Within the black module, module membership was tightly correlated with gene significance for the trait (cor = 0.84, p = 1.4 × 10⁻²^8^), confirming that the most central module genes were also the most disease-relevant (**Figure 2C**). The 103 genes constituting this module were therefore designated as PF-related module genes. Intersecting these 103 module genes with the 406 DEGs yielded 55 high-confidence PF-related genes for downstream analysis (**Figure 2E**). Notably, under this trait encoding, the module eigengene correlated positively with PF while several member genes such as *MT2A* were upregulated, consistent with co-expression module characteristics.

**Figure 2:**
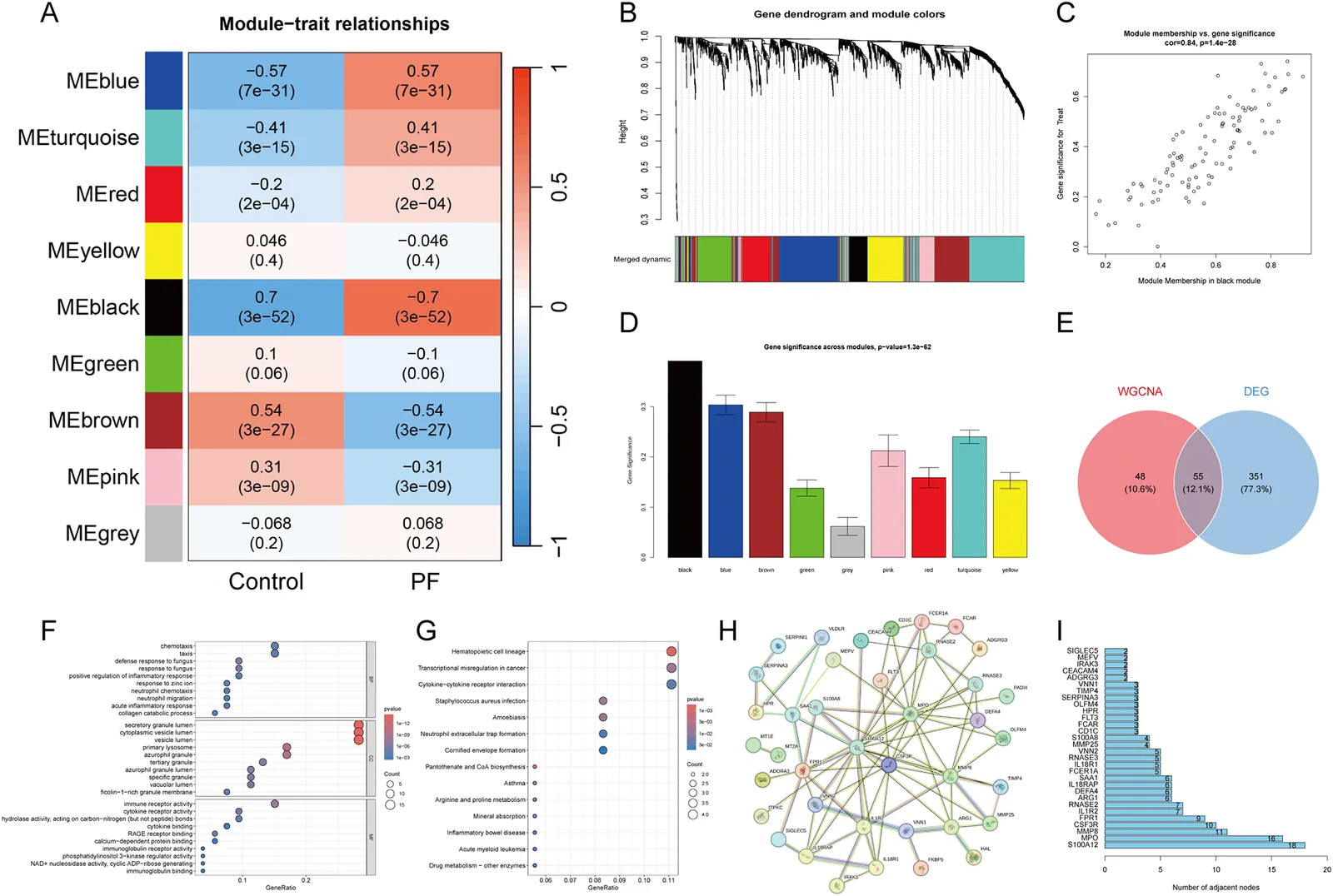
WGCNA, functional enrichment, and PPI analysis of PF-related genes. **(A)** Module–trait relationship heatmap; each cell shows the correlation coefficient (p-value). The black module (MEblack) was most strongly associated with disease status (|cor| = 0.70 for Control/PF, p = 3 × 10^-52^). **(B)** Gene clustering dendrogram with assigned module colors. **(C)** Module membership versus gene significance in the black module (cor = 0.84, p = 1.4 × 10⁻²^8^). **(D)** Mean gene significance across modules. **(E)** Venn diagram of WGCNA black-module genes and DEGs, yielding 55 shared PF-related genes. **(F)** GO and **(G)** KEGG enrichment of the 55 genes; dot size, gene count; color, adjusted p-value. **(H)** PPI network (STRING/Cytoscape). **(I)** Top hub nodes ranked by degree, with S100A12 and MPO as the dominant hubs.

Functional enrichment of these 55 genes revealed a coherent innate-immune and chemotactic program. GO analysis showed enrichment in biological processes including chemotaxis, acute inflammatory response and defense response to fungus; cellular components localized to secretory granules and the granule lumen; and molecular functions centered on immune receptor activity and cytokine binding (**Figure 2F**). KEGG analysis indicated enrichment in immune-related pathways such as haematopoietic cell lineage, cytokine–cytokine receptor interaction, neutrophil extracellular trap formation and *Staphylococcus aureus* infection, alongside pantothenate and CoA biosynthesis (**Figure 2G**). The PPI network displayed a scale-free topology with several highly connected hub nodes and dense intra-module connectivity (**Figure 2H**). Ranking nodes by degree identified S100A12 (degree = 18) and MPO (degree = 16) as the two dominant interaction hubs (**Figure 2I**).

### 3.3 Machine learning and SHAP analysis

We utilized a total of 112 ML algorithms to develop PF diagnostic and predictive models, assessing their efficacy on separate cohorts. **Figure 3A** illustrates that all models were trained on our primary dataset and then verified on cohorts GSE47460A and GSE47460B. The results indicate that the LASSO model had exceptional and consistent predictive performance, attaining the greatest AUC on the training set (AUC = 0.994) and sustaining strong accuracy in external validation (AUCs of 0.821 and 0.816, respectively). Through L1 regularization, LASSO yields sparse solutions that automatically select features and reduce overfitting, producing a concise, interpretable set of predictors well suited to downstream SHAP analysis. The model was thus validated both by robust performance on an independent cohort and by SHAP-based interpretability of its decision logic. 15 hub genes were involved in LASSO model construction: *ADM, CDA, CRIP1, FCER1A, GCA, HAL, IL18R1, IRAK3, IRS2, MPO, MT2A, SENP7, SERPINA3, SLCO4A1, TMEM2* (**Figures 3B, C**). The violin plot shows that higher expression of *MT2A*, *CRIP1*, *FCER1A*, *TMEM2*, *SENP7*, *IRAK3*, and CDA is associated with higher SHAP values, indicating that these features push the model’s prediction toward the fibrotic label, whereas *SLCO4A1, IL18R1, MPO, ADM, IRS2, GCA, HAL*, and *SERPINA3* push it toward the control label. These are directional contributions to the classifier, not evidence that the genes themselves cause or protect from disease (**Figure 3B**). These candidates were further validated in bulk transcriptomes and by knockdown and overexpression experiments. The bar plot illustrates the SHAP values of the genes included in the model, with *MT2A, CRIP1, SLCO4A1*, and *IL18R1*, ranking as the top four genes based on SHAP value (**Figure 3C**). We designate the four most significant genes as key genes. **Figure 3D** illustrates that all four hub genes exhibited diagnostic discrimination capability, with the AUC above 0.70. The SHAP dependence scatter plot (**Figure 3E**) shows that *MT2A* and *CRIP1* exert an increasingly positive contribution to the model’s prediction as their expression rises, whereas *SLCO4A1* and *IL18R1* exert an increasingly negative contribution.

**Figure 3:**
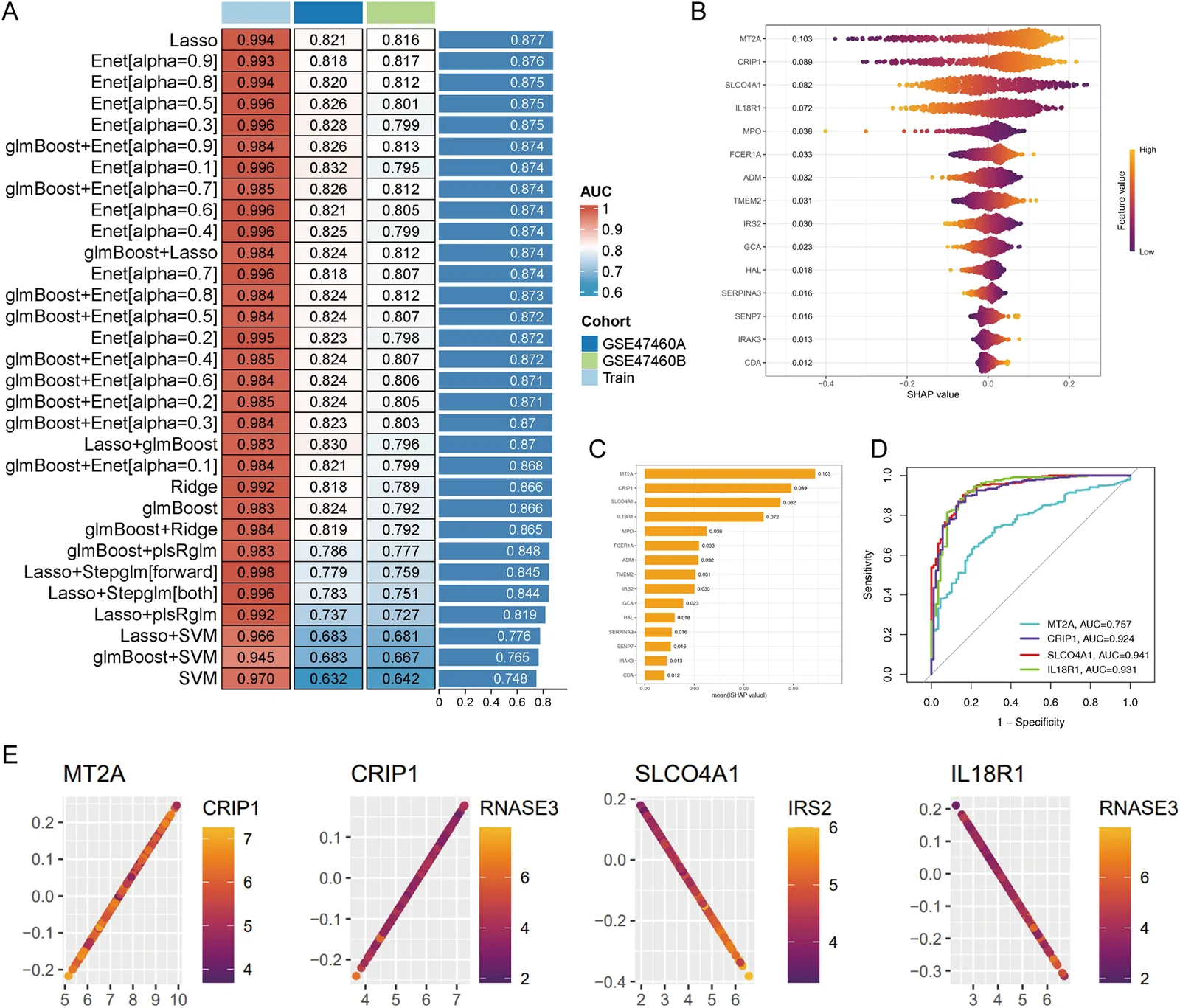
Identification and validation of hub genes in PF based on ML and SHAP analysis. **(A)** Heatmap compares the AUC of various ML models to determine the best predictive model (LASSO). **(B)** Violin plot depicts the expression distribution of core genes in PF and the control group. Higher expression of *MT2A* and *CRIP1* is associated with higher SHAP values, whereas *SLCO4A1* and *IL18R1* show the opposite. **(C)** SHAP analysis results for the intersecting genes, shown as a SHAP value bar chart. **(D)** ROC curves validate the independent data for core genes. **(E)** SHAP dependence scatter plot visualizes the contribution of genes to the LASSO model.

### 3.4 Single-cell RNA-seq analysis

We performed scRNA-seq analysis to delineate the circulating immune cell composition and transcriptional states in PF patients (21).Circulating immune cells are recruited to the fibrotic lung and have been linked to disease progression and outcome in PF. The peripheral immune compartment is therefore a relevant setting in which to assess the cellular expression of the key genes. The number of genes detected per cell scaled closely with sequencing depth (r = 0.95; **Supplementary Figure 1A**). The mitochondrial read fraction showed only a weak inverse correlation with depth (r = −0.28; **Supplementary Figure 1B**). The top 10 highly variable genes underpinning cellular heterogeneity were identified (**Supplementary Figure 1C**). Unsupervised clustering via the tSNE algorithm revealed multiple distinct cell populations, visualized with cluster-specific coloration (**Supplementary Figure 1D**). Robust cluster-specific marker genes were identified, with their expression percentage and average level summarized in a bubble plot (**Supplementary Figure 1E**). These were annotated as CD4^+^ T cells, CD8^+^ T cells, NK cells, B cells, CD14^+^ classical monocytes, CD16^+^ non-classical monocytes, dendritic cells, proliferating cells, platelets, and hematopoietic stem/progenitor cells (HSPCs), based on canonical marker expression (**Supplementary Figure 1E**). All ten populations were represented in both control and PF samples (**Supplementary Figure 1F**).

We next projected the four key genes onto the t-SNE embedding (**Supplementary Figures 1G–J**). *MT2A* and *CRIP1* were robustly expressed across the major immune lineages, including monocytes and lymphocytes (**Supplementary Figures 1G, H**). By contrast, *SLCO4A1* and *IL18R1* were detected only in sporadic cells at near-background levels across all populations (**Supplementary Figures 1I, J**). The two genes that ranked highest by SHAP value and were positively associated with PF in the LASSO model were thus also the most robustly expressed at scRNA-seq. The two negatively associated predictors, in contrast, were scarcely detectable. On the basis of this concordance between model contribution and cellular expression, we prioritized *MT2A* as a core gene for subsequent analyses.

This scRNA-seq dataset served for cell-type-resolved target nomination and VGKO-based prediction of downstream effectors. *MT2A* encodes a ubiquitously induced cysteine-rich stress-response protein, so its detection in circulating immune cells is compatible with its induction in injured lung epithelium; the whole-lung bulk transcriptomes used for ML training already show *MT2A* upregulation in fibrotic parenchyma. Because epithelial EMT is a central mechanism of fibrotic remodeling, we tested the predicted MT2A-S100A8 axis in TGF-β1-stimulated BEAS-2B cells.

### 3.5 *MT2A* virtual gene knockout and downstream pathway analysis

Virtual knockdown of *MT2A* using scTenifoldKnk revealed a highly specific transcriptional response, with the gene encoding *S100A8* emerging as the most significantly perturbed target (30, 41). *S100A8* expression was perturbed (p < 10⁻¹^5^), markedly exceeding all other DEGs (**Figure 4A**). Global analysis indicated that only 1.8% of profiled genes were significantly altered, demonstrating that *MT2A* knockdown induces a focused transcriptional reprogramming (**Figure 4B****).** The volcano plot also displays *S100A8*’s greatest disturbance both before and after *MT2A* knockdown (**Figure 4C**). In bulk transcriptomic data (pooled GSE17978, GSE32537, GSE40839 and GSE53845), both *MT2A* (P = 0.0238) and S100A8 (P = 0.0068) were upregulated in the PF group (**Figure 4D**). The coordinated upregulation of MT2A and S100A8 in lung parenchyma corroborates the *MT2A-S100A8* prediction derived from PBMC-derived VGKO and provides the tissue-level rationale for testing this axis in bronchial epithelial cells. GSEA analysis of DEGs revealed substantial enrichment in inflammatory and immunological regulatory networks, notably within the chemokine and cytokine-cytokine receptor interaction pathways (such as CCL2, CXCL8, TNF, IL6). This pattern indicates that *MT2A* may serve as a regulator of innate immunological and inflammatory signaling, with S100A8 as a key downstream effector (**Figure 4E**).

**Figure 4:**
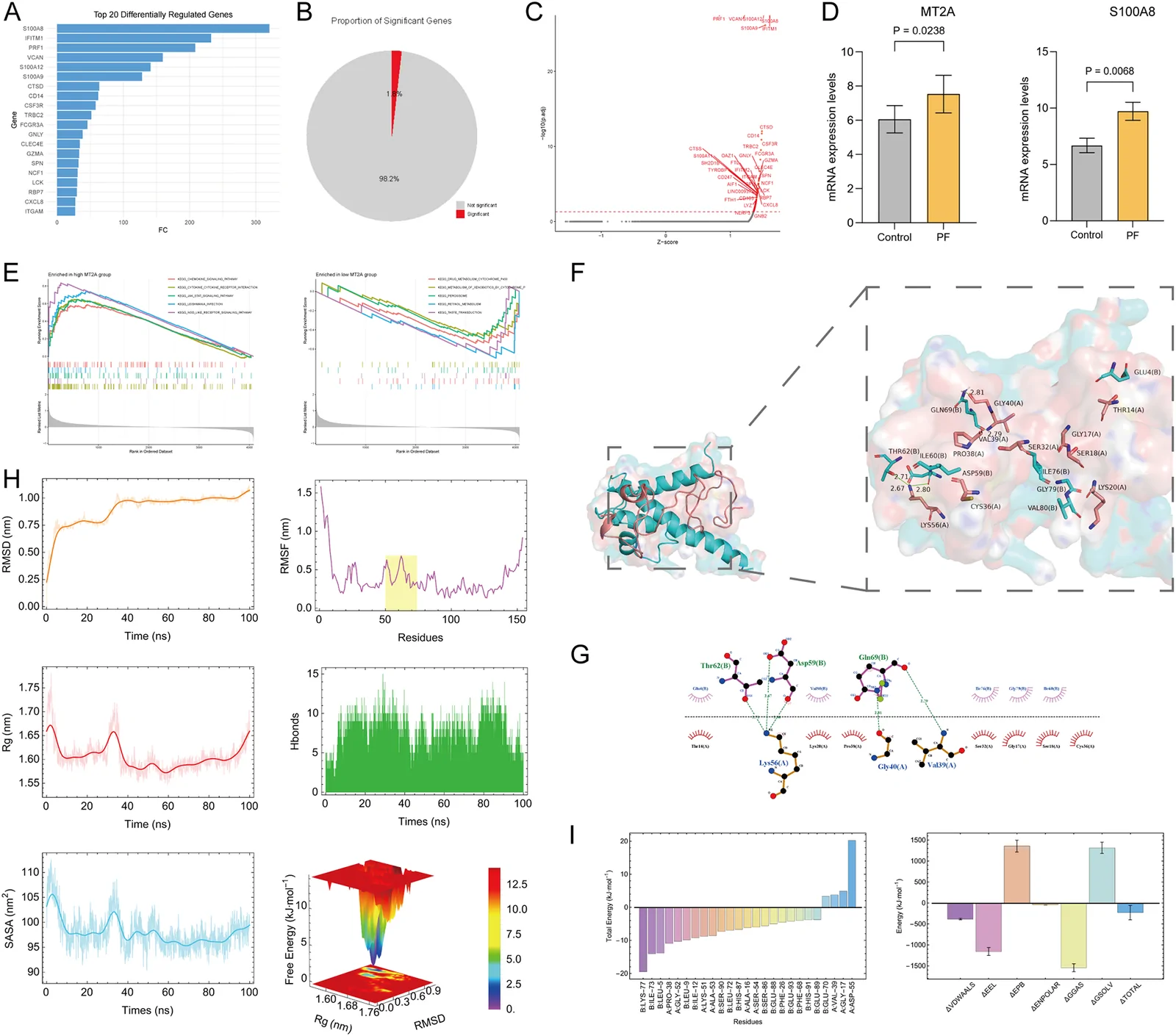
*MT2A* virtual gene knockout and downstream pathway analysis. **(A)** Changes in downstream gene expression after *MT2A* gene knockout. *S100A8* was the most disturbed gene. **(B)** Pie plot. Red represents the proportion of significant DEGs. **(C)** Volcano plot. The X-axis represents the Z-score and the Y-axis represents the adjusted p-value, red dots represent significantly DEGs. **(D)** The expression levels of *MT2A* and *S100A8* in bulk transcript data. **(E)** *MT2A* was enriched in the GSEA pathway in the high and low expression groups. **(F)** Spatial arrangement of the MT2A-S100A8 protein complex. **(G)** Illustration of the interface between MT2A and S100A8. **(H)** Analysis of the MD stability of the MT2A-S100A8 protein complex, including RMSD, RMSF, Rg, hydrogen bonds, SASA, and Gibbs FEL. **(I)** MM-PBSA results for the MT2A-S100A8 protein complex, showing residue contribution analysis and statistics on binding free energy components.

The spatial (**Figure 4F**) and planar (**Figure 4G**) positions of protein-protein docking in MT2A-S100A8 are displayed. As shown in **Figure 4H**, the RMSD of the MT2A-S100A8 complex stabilized over the 100 ns simulation (fluctuations ≈1.05 nm), with a high RMSF in residues 50–75 indicating interfacial flexibility, and the radius of gyration stabilizing near 1.66 nm. Interfacial hydrogen bonds remained stable between 4 and 10 throughout the trajectory, and a minimum-energy cluster formed in the Gibbs free energy landscape (FEL). MM-PBSA calculations on the final 10 ns of the equilibrated trajectory gave an average binding free energy of –229.5 kJ/mol for the MT2A-S100A8 complex. MM-PBSA neglects configurational entropy and treats electrostatics in a continuum solvent, so this value is not on the same scale as an experimental free energy of binding. We use it only to confirm that the interface is energetically favorable and to rank per-residue contributions. The analysis of per-residue energy contributions revealed that A: PRO-38 and A: GLY-52 of MT2A, together with B: LYS-77 and B: ILE-73 of S100A8, are critical stabilizing residues. Their significant negative energy contributions establish the structural foundation for the stability of the complex (**Figure 4I**).

### 3.6 AI-driven drug screening and analysis with DrugReflector

In AI-driven drug screening, BRD-K54403101 achieved the highest rank in the candidate library (logit = 9.794), signifying strong association with the target disease model (**Table 2**; **Supplementary Figure 2A**). The three-dimensional structure of this chemical was elucidated (**Supplementary Figure 2B**), affirming its capacity to establish a stable complex with the target protein MT2A (**Supplementary Figure 2C**).

**Table 2:** AI-driven drug screening by DrugReflector.

Rank	Drugs	Logit	Prob
1	BRD-K54403101	9.793996	0.013957328
2	BRD-K44575778	9.7816105	0.013785527
3	BRD-K44494044	9.405847	0.009467411
4	BRD-K40530731	9.349093	0.008945067
5	BRD-K53874318	9.337596	0.008842811
6	BRD-K71003802	9.327905	0.008758
7	BRD-K41520144	9.315586	0.00865
8	BRD-K44241590	9.153912	0.007359
9	BRD-K14807180	9.143952	0.007286
10	BRD-K47700511	9.131557	0.007196

MD confirmed conformational stability: RMSD, RMSF and hydrogen-bond count remained stable throughout the trajectory (**Supplementary Figure 2D**). MM-PBSA decomposition gave a binding free energy of ΔG = -130.5 kJ/mol and showed that binding was dominated by van der Waals and electrostatic contributions, identifying the residues that account for most of the interaction energy (**Supplementary Figure 2E**). BRD-K54403101 is therefore a computationally prioritized starting point for chemical optimization.

### 3.7 MT2A and S100A8 co-exist in a common complex in transfected cells

Reciprocal co-immunoprecipitation of Flag-S100A8 and HA-MT2A expressed in HEK293T cells recovered HA-MT2A in anti-Flag immunoprecipitates (**Figure 5A**) and Flag–S100A8 in anti-HA immunoprecipitates (**Figure 5B**), with no signal in isotype-matched IgG controls. This assay establishes that the two overexpressed, epitope-tagged proteins co-exist in a common complex in cells; it does not by itself demonstrate direct binding, which we therefore addressed independently by surface plasmon resonance.

**Figure 5:**
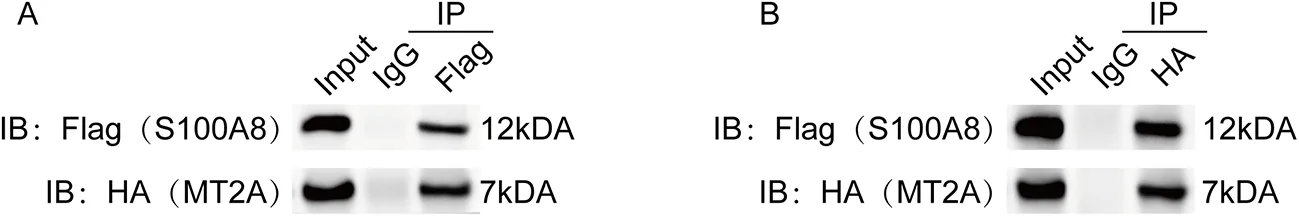
Reciprocal co-immunoprecipitation of Flag-S100A8 and HA-MT2A co-expressed in HEK293T cells. **(A)** Immunoprecipitation with anti-Flag or normal mouse IgG. **(B)** Immunoprecipitation with anti-HA or normal rabbit IgG. Input, 10% of lysate.

### 3.8 Surface plasmon resonance

We measured the S100A8-MT2A interaction by SPR, injecting S100A8 at 7.5, 15, 30, 62.5, 125 and 250 nM over immobilized MT2A (**Figure 6A**). The equilibrium response increased in a concentration-dependent manner, from 2.0 RU at 7.5 nM to 18.1 RU at 250 nM, and reached 77% of the fitted maximal response at the highest concentration tested; the interaction therefore approached, but did not reach, saturation within the 180 s injection (**Figure 6B**). Fitting the steady-state responses to a 1:1 binding model gave a dissociation constant (Rmax = 23.4 ± 2.3 RU, R² = 0.98), which described the data better than a non-saturable linear model (R² = 0.91). Responses were largely free of bulk refractive-index contributions and 68-78% of the plateau signal persisted at the end of the dissociation phase, indicating that binding was reversible. Because the highest concentration reached only 77% of Rmax, the fitted KD derives partly from extrapolation; the measurement was performed in one orientation and in a single experiment, and we therefore treat 88 nM as evidence of sub-micromolar affinity.

**Figure 6:**
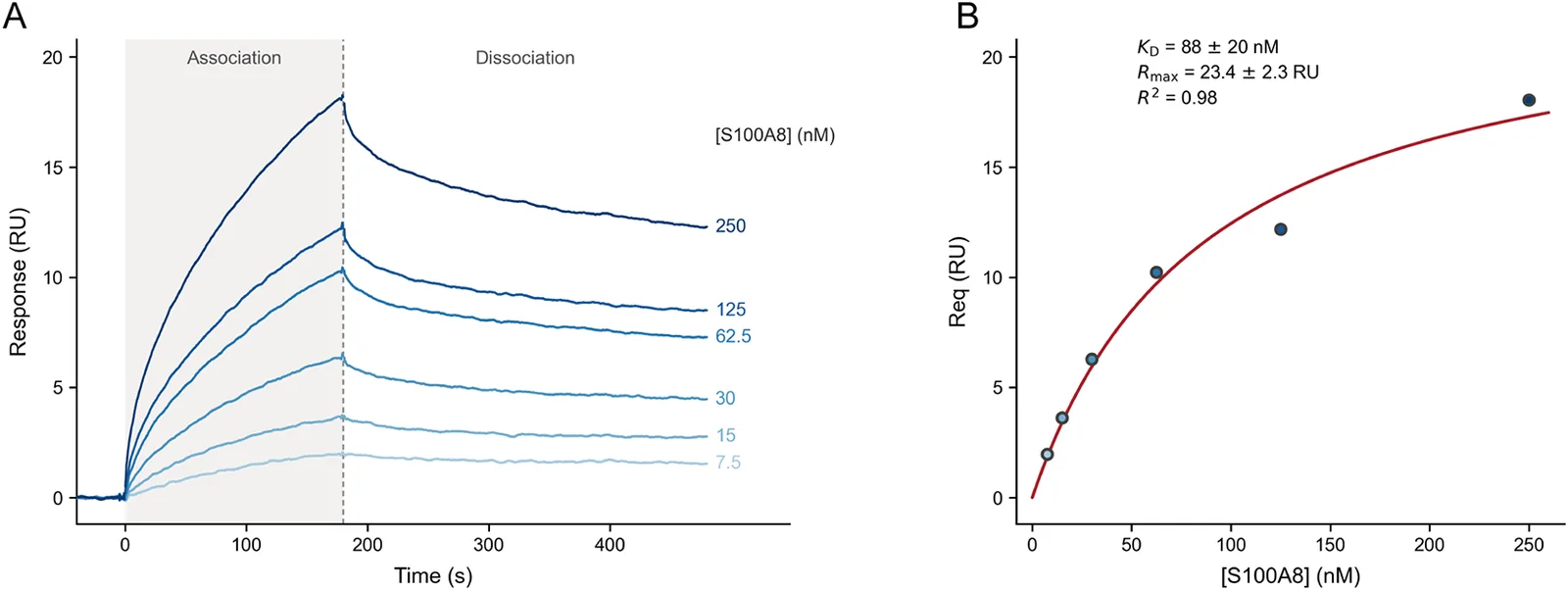
SPR analysis of the MT2A-S100A8 interaction. **(A)** Overlaid reference-subtracted sensorgrams at six S100A8 concentrations (7.5, 15, 30, 62.5, 125 and 250 nM), with 180 s association and 300 s dissociation. **(B)** Steady-state response as a function of S100A8 concentration, fitted to a 1:1 binding model; KD = 88 ± 20 nM, Rmax = 23.4 ± 2.3 RU (R² = 0.98). The highest concentration reached 77% of Rmax. Data are from a single representative experiment.

### 3.9 Bleomycin induces severe PF and a prominent inflammatory response in mice

To evaluate the pathological alterations induced by BLM, we conducted a comprehensive histological analysis of lung tissues. H&E staining revealed that, compared to the control group, BLM-treated mice exhibited severe disruption of the alveolar architecture, significant thickening of the alveolar septa, and extensive inflammatory cell infiltration (**Figure 7A**). Masson’s trichrome and Sirius Red staining further confirmed massive collagen deposition within the pulmonary interstitium in the BLM group (**Figure 7A**). Quantitative analyses demonstrated a significant elevation in both the AST and CVF, CVF_SR following BLM administration (**Figure 7B**).

**Figure 7:**
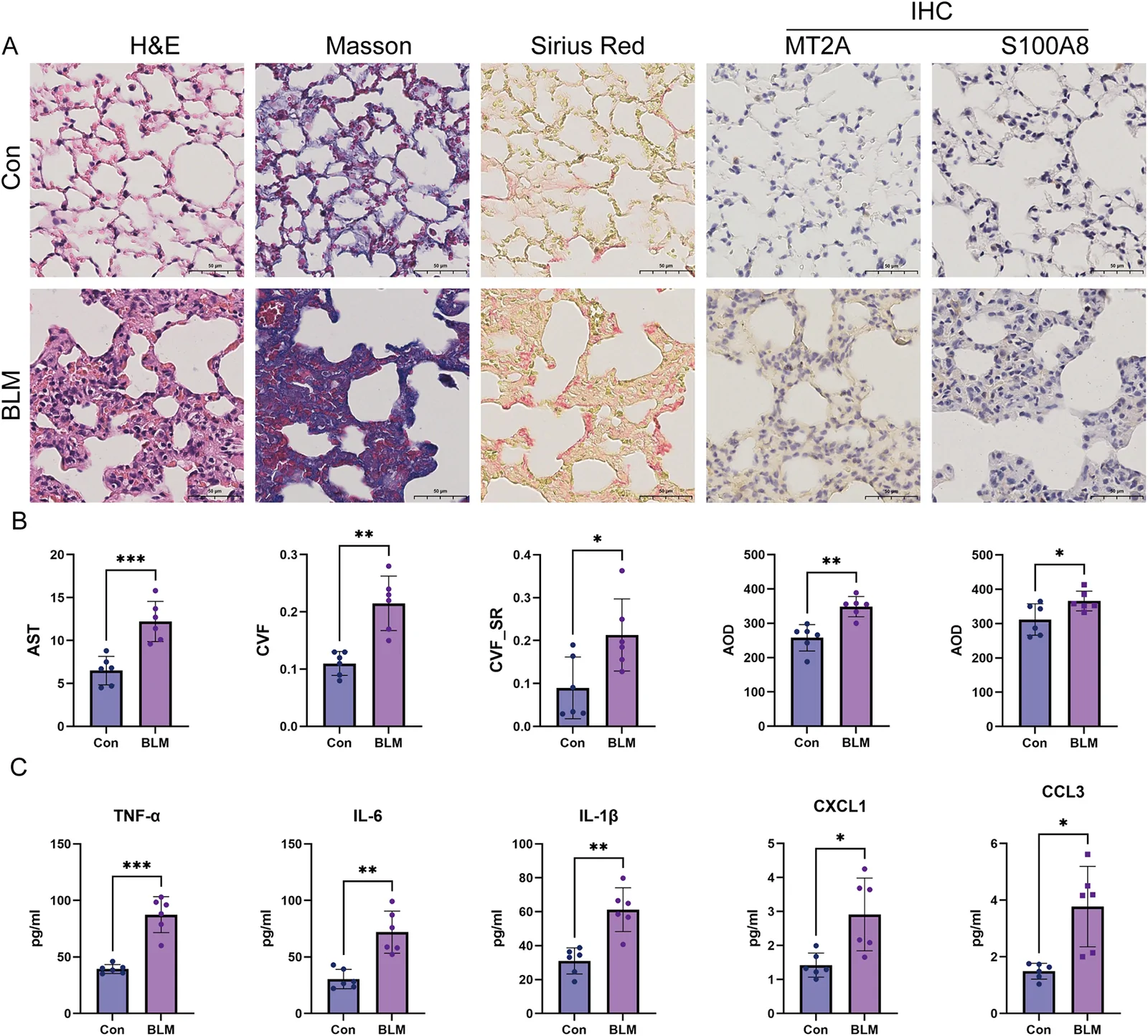
BLM induces severe PF and prominent inflammatory responses in mice. **(A)** Representative histological and IHC images of lung tissues from the control and BLM groups. Sections were evaluated using H&E, Masson’s trichrome, and Sirius Red staining, alongside IHC staining for MT2A and S100A8. Scale bars, 50 μm. **(B)** Quantitative assessment of PF and target protein expression. Bar graphs show the AST, CVF, CVF_SR, and AOD for MT2A and S100A8 IHC staining. **(C)** Secretion levels of pro-inflammatory cytokines (TNF-α, IL-6, IL-1β, CXCL1 and CCL3) in serum, measured in pg/ml. Data are presented as mean ± SD (n = 6); *P < 0.05, **P < 0.01, ***P < 0.001.

Concurrently, immunohistochemical profiling revealed that the expression levels of MT2A and S100A8 were significantly upregulated in the heavily fibrotic lung tissues (**Figures 7A, B**). Given that profound tissue remodeling is typically driven by immune activation, we next assessed the inflammatory milieu. BLM treatment provoked a robust inflammatory cascade, characterized by a statistically significant surge in the secretion of pro-inflammatory cytokines (TNF-α, IL-6, IL-1β, CXCL1 and CCL3) compared to the controls (**Figure 7C**). Collectively, these data demonstrate that BLM successfully induces a pathology characteristic of PF accompanied by extensive inflammatory mediator release.

### 3.10 TGF-β1 induces the expression and cytoplasmic localization of MT2A and S100A8 in BEAS-2B cells

To define an optimal TGF-β1 concentration for modeling EMT, BEAS-2B cells were exposed to graded TGF-β1 (0–30 ng/ml) and assessed by CCK-8. At both 24 h and 48 h, relative viability remained essentially unchanged across the 0–15 ng/ml range (ns versus untreated control) and declined significantly only at concentrations ≥20 ng/ml (24 h, P < 0.01; 48 h, P < 0.05) (**Figure 8A**), indicating that TGF-β1 up to 15 ng/ml is well tolerated whereas higher doses are growth-suppressive. In parallel, Annexin V–FITC/PI flow cytometry showed that the apoptotic fraction (early + late, Q2 + Q3) was low and statistically comparable between 0 and 10 ng/ml (~7%, ns) but rose significantly at 15 ng/ml (~17%) and further at 30 ng/ml (~19%) (**Figure 8B**). Thus, 15 ng/ml was the highest concentration that preserved overall cell viability while reliably engaging a stress/EMT response; although apoptosis was modestly increased at this dose, the large majority of cells remained viable, and 15 ng/ml was therefore selected for all subsequent EMT experiments.

**Figure 8:**
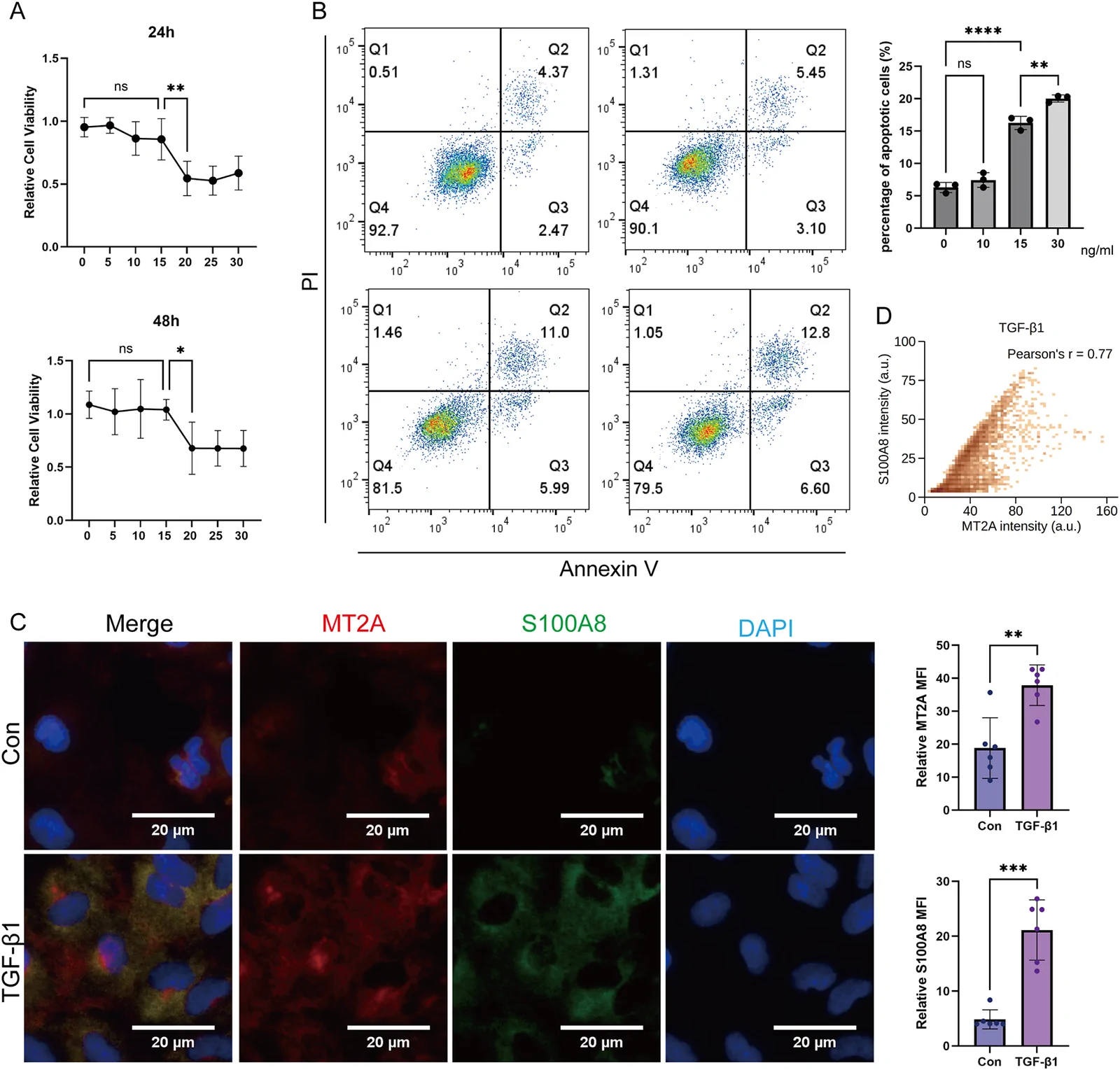
TGF-β1 induces the upregulation and cytoplasmic localization of MT2A and S100A8 in BEAS-2B cells. **(A)** CCK-8 cell-viability assay of BEAS-2B cells treated with graded concentrations of TGF-β1 (0–30 ng/ml) for 24 h and 48 h; relative viability was maintained up to 15 ng/ml and decreased significantly at concentrations ≥20 ng/ml. **(B)** Annexin V-FITC/PI flow-cytometric analysis of apoptosis in cells exposed to increasing TGF-β1 (0, 10, 15 and 30 ng/ml), with quantification of the total apoptotic fraction (Annexin V^+^ cells). **(C)** Immunofluorescence staining of MT2A and S100A8 in BEAS-2B cells, with nuclei counterstained by DAPI; cytoplasmic co-localization is evident in the merged images and was quantified by Pearson’s correlation coefficient. Scale bar, 20 μm. **(D)** Quantification of mean fluorescence intensity of MT2A and S100A8. Data are presented as mean ± SD (n = 3); *P < 0.05, **P < 0.01, ***P < 0.001, ****P < 0.0001.

To examine the expression and subcellular localization of MT2A and S100A8 in TGF-β1-stimulated airway epithelial cells, we performed double immunofluorescence staining in BEAS-2B cells (**Figure 8C**). Under basal conditions, MT2A was weakly expressed and S100A8 was barely detectable. TGF-β1 treatment increased the fluorescence intensity of MT2A (P < 0.01) and that of S100A8 (P < 0.001). Both proteins localized predominantly to the cytoplasm, with partial perinuclear enrichment, and showed extensive signal overlap in merged images. Colocalization analysis yielded a Pearson’s correlation coefficient of 0.75 ± 0.17 (**Figure 8D**). These data indicate that TGF-β1 coordinately induces MT2A and S100A8 and promotes their colocalization in the cytoplasm of epithelial cells.

### 3.11 *MT2A* knockdown suppresses TGF-β1-induced pro-fibrotic responses in BEAS-2B cells

TGF-β1 stimulation significantly enhanced the migration, invasion, and proliferation of BEAS-2B cells. However, these TGF-β1-induced pathogenic cellular behaviors were significantly suppressed by siRNA-mediated *MT2A* gene knockdown (**Figure 9**). These results indicate that *MT2A* is not merely a disease-related biomarker but a functional regulator downstream of TGF-β1 that supports epithelial activation, migration and proliferation, identifying it as a candidate therapeutic target that requires *in vivo* validation.

**Figure 9:**
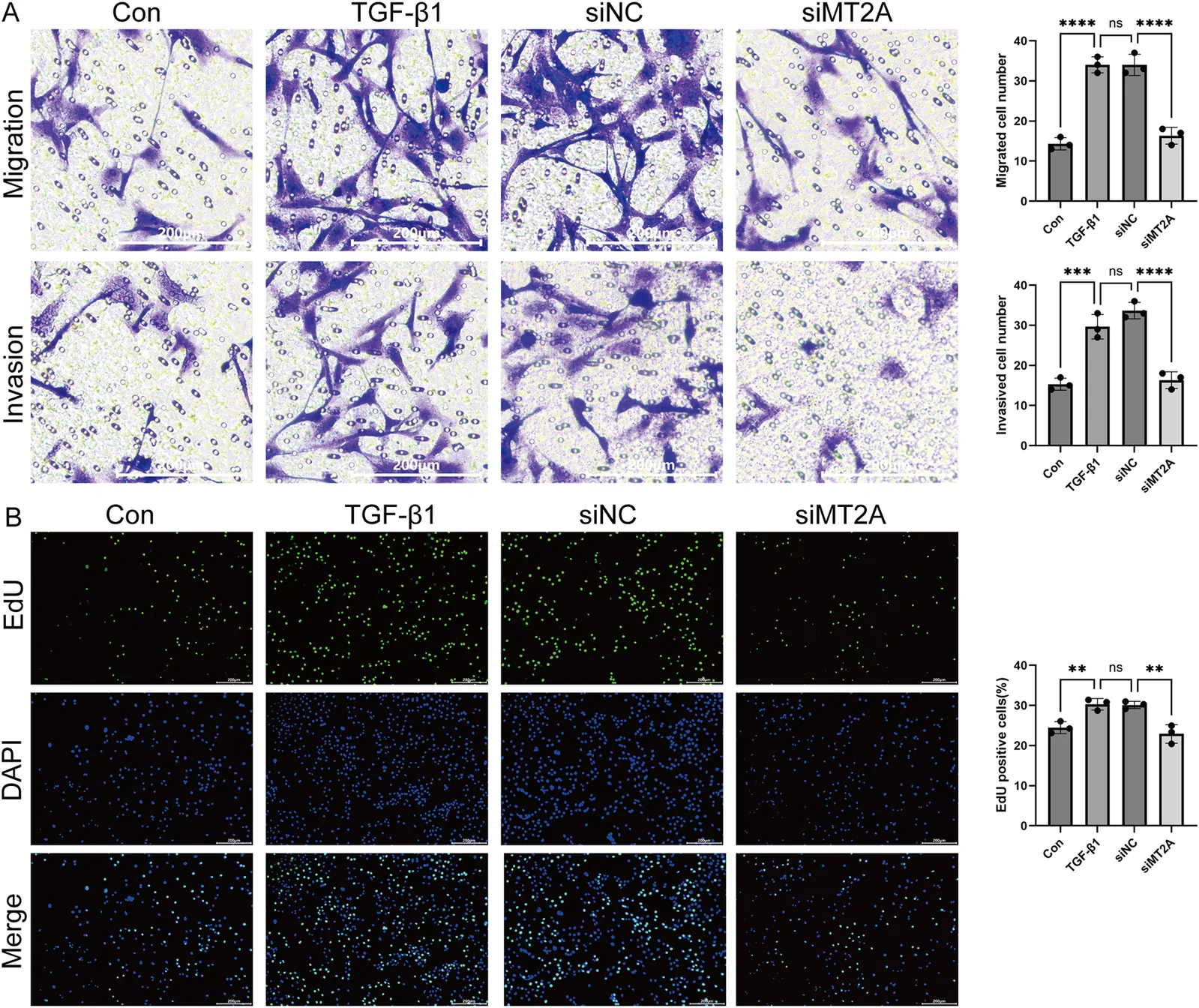
*MT2A* knockdown attenuates TGF-β1-induced migration, invasion, and proliferation of BEAS-2B cells. **(A)**​ Transwell assays demonstrating that TGF-β1 stimulation promotes the migration and invasion of BEAS-2B cells, an effect that is significantly attenuated by siRNA-mediated knockdown of *MT2A* (si*MT2A*). Scale bar, 100μm. **(B)**​ EdU proliferation assays showing that TGF-β1 enhances the proliferation of BEAS-2B cells, which is markedly suppressed upon *MT2A* knockdown. Scale bar, 50μm. Data are presented as mean ± SD (n = 3); **P < 0.01, ***P < 0.001, ****P < 0.0001.

We further investigated the molecular mechanism of MT2A in the TGF-β1-induced profibrotic phenotype of BEAS-2B cells. TGF-β1 treatment not only significantly upregulated the mRNA and protein expression of MT2A itself, but also induced the upregulation of its predicted interacting protein S100A8, driving the EMT process. Specifically, this manifested as the upregulation of COL1A1, VIM and FN1 expression, while the expression of the *CDH1*/E-cad decreased. Notably, knockdown of *MT2A* effectively reversed these molecular changes. In cells where *MT2A* was suppressed, the expression of TGF-β1-induced EMT-related genes (COL1A1, VIM, FN1) was significantly inhibited, while *CDH1*/E-cad expression was partially restored. Crucially, the knockdown of *MT2A* led to a decrease in the expression of S100A8 at both the mRNA and protein levels (**Figure 10**). This result is consistent with our bioinformatics prediction that S100A8 lies downstream of MT2A and provides experimental support for a directional MT2A-to-S100A8 relationship; the level at which MT2A acts on S100A8 is addressed below.

**Figure 10:**
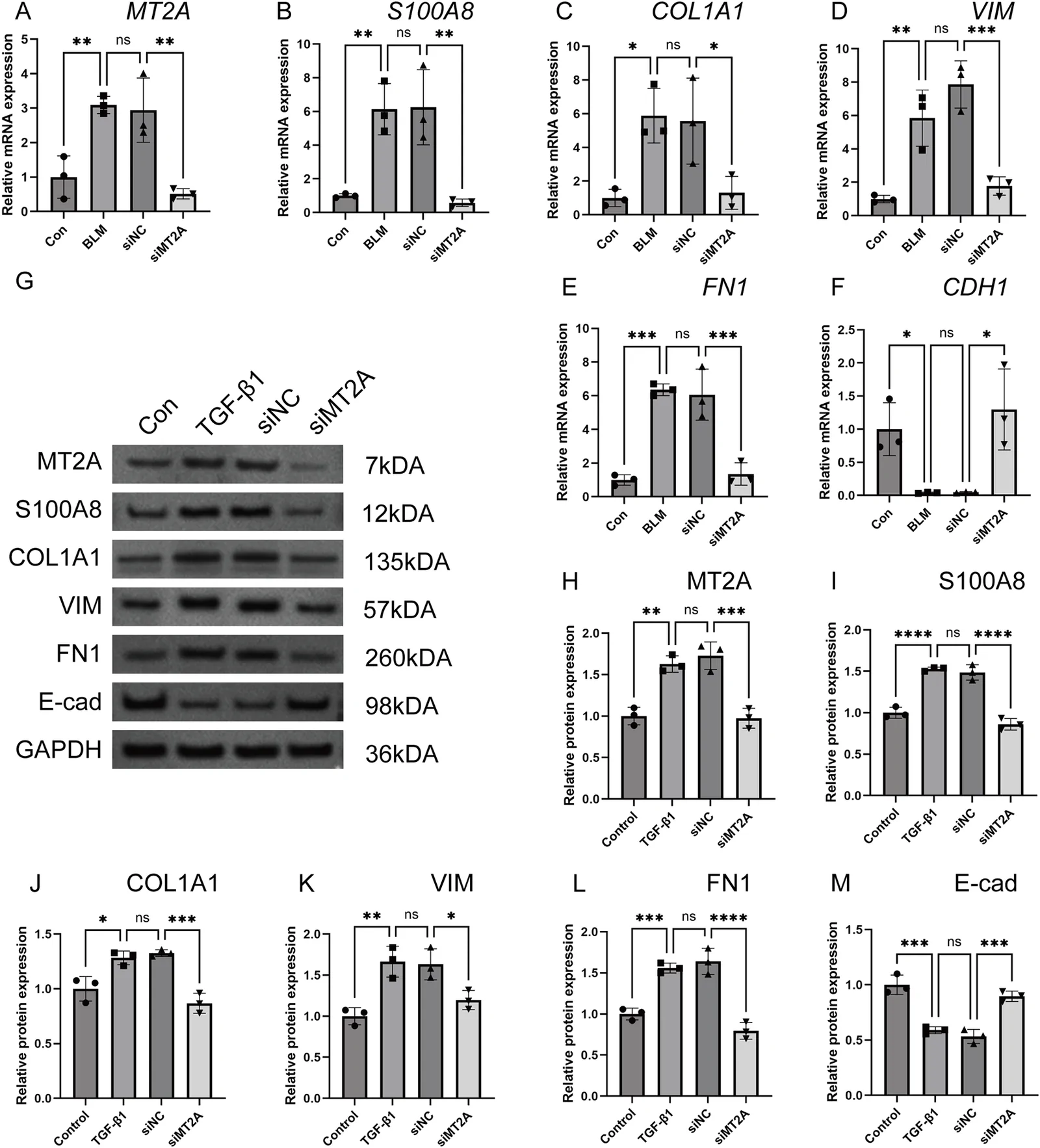
*MT2A* knockdown inhibits TGF-β1-induced expression of MT2A/S100A8 and EMT in BEAS-2B cells. **(A–F)**​ qRT-PCR analysis of the relative mRNA expression levels of *MT2A* **(A)**, *S100A8* **(B)**, *COL1A1* **(C)**, *VIM* **(D)**, *FN1* **(E)**, and *CDH1* **(F)** in BEAS-2B cells. **(G)**​ Western blotting images and quantitative analysis of protein levels for MT2A/S100A8 and EMT in BEAS-2B cells. **(H–M)**​ Western blotting analysis of the relative protein expression levels of MT2A **(H)**, S100A8 **(I)**, COL1A1 **(J)**, VIM **(K)**, FN1 **(L)**, and E-cad **(M)**. Data are presented as mean ± SD (n = 3); *P < 0.05, **P < 0.01, ***P < 0.001, ****P < 0.0001.

### 3.12 *S100A8* re-expression restores the EMT program in MT2A-depleted cells

To establish whether S100A8 is the mediator of MT2A-driven EMT, we performed a rescue experiment in which *S100A8* was re-expressed in *MT2A*-depleted, TGF-β1-stimulated BEAS-2B cells. *S100A8* overexpression left *MT2A* unchanged at both the mRNA and protein level in siNC- and si*MT2A*-transfected cells (**Figures 11A, G, H**), confirming that S100A8 operates downstream of MT2A without feedback onto it. In *MT2A*-knockdown cells, forced *S100A8* expression restored S100A8 to levels indistinguishable from siNC+OE-*S100A8* controls (**Figures 11B, I**) and fully reinstated the EMT program: COL1A1, VIM and FN1 were re-induced at both the transcript and protein level, whereas *CDH1*/E-cad was again suppressed (**Figures 11C–F, J–M**). For every marker examined, the si*MT2A*+OE-*S100A8* and siNC+OE-*S100A8* groups were statistically indistinguishable, indicating complete rescue. MT2A is therefore dispensable for EMT once S100A8 is supplied, placing S100A8 downstream of MT2A as the necessary effector through which MT2A licenses TGF-β1-induced EMT.

**Figure 11:**
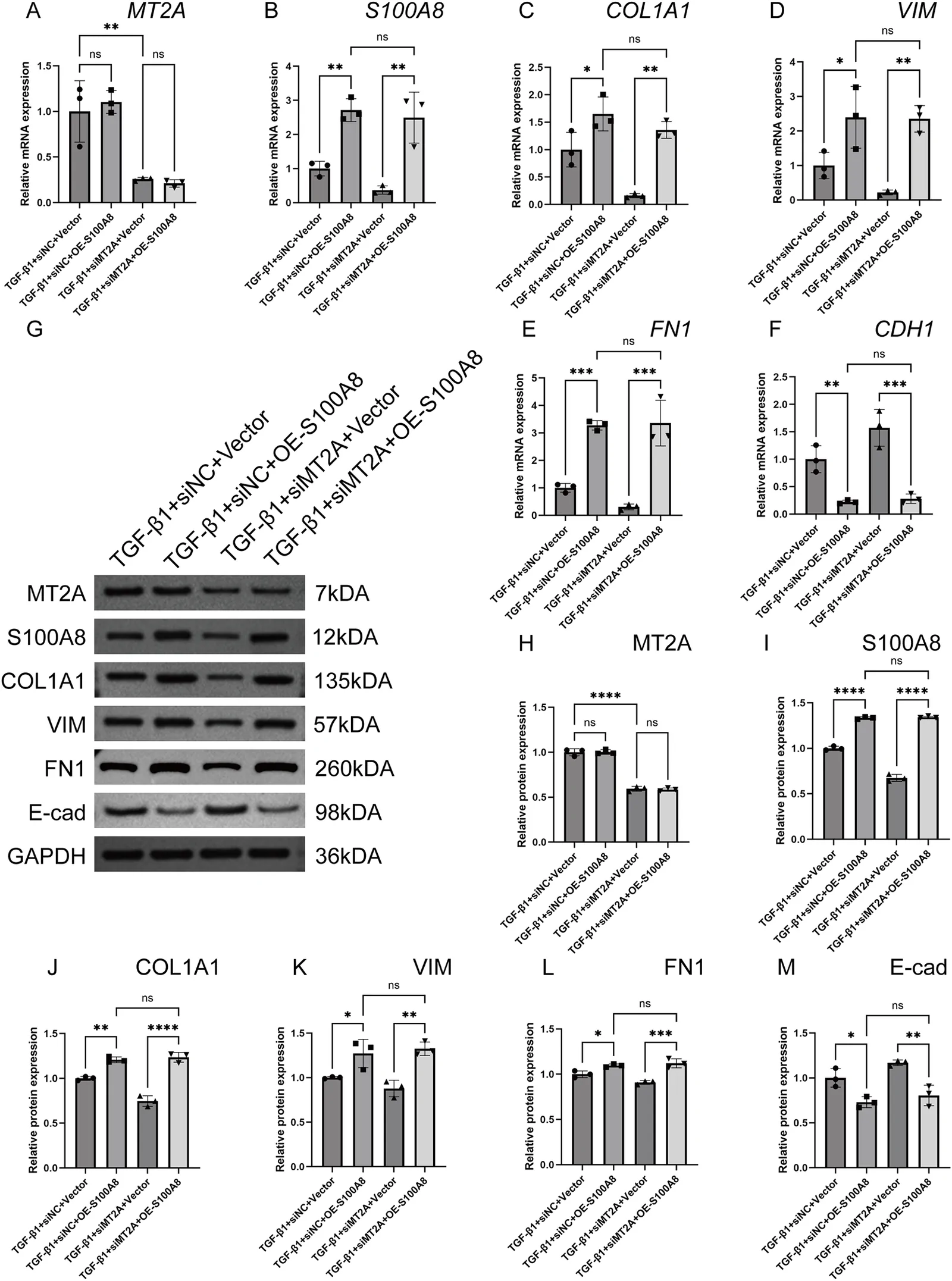
*S100A8* re-expression rescues the EMT program in MT2A-depleted BEAS-2B cells. Cells were co-transfected with siMT2A or siNC together with an OE-*S100A8* or the corresponding empty vector, and stimulated with TGF-β1 (15 ng/ml) for 48 h. **(A–F)** qRT-PCR analysis of the relative mRNA levels of *MT2A* **(A)**, *S100A8* **(B)**, *COL1A1* **(C)**, *VIM* **(D)**, *FN1* **(E)** and *CDH1* **(F)**, normalized to GAPDH. **(G)** Representative immunoblots of MT2A, S100A8, COL1A1, VIM, FN1 and E-cad, with GAPDH as loading control. **(H–M)** Densitometric quantification of MT2A **(H)**, S100A8 **(I)**, COL1A1 **(J)**, VIM **(K)**, FN1 **(L)** and E-cad **(M)**, normalized to GAPDH and expressed relative to the TGF-β1+siNC+Vector group. Data are presented as mean ± SD (n = 3); *P < 0.05, **P < 0.01, ***P < 0.001, ****P < 0.0001.

### 3.13 *MT2A* overexpression promotes TGF-β1-induced pro-fibrotic responses in BEAS-2B cells

To test whether MT2A is sufficient to promote the pro-fibrotic phenotype, we conducted gain-of-function experiments. As shown in **Figure 12**, overexpression of *MT2A* (OE-*MT2A*) in BEAS-2B cells further amplified the pro-fibrotic effect of TGF-β1. Compared with the empty vector control group, overexpression of *MT2A* significantly enhanced TGF-β1-induced cell migration, invasion, and proliferation (**Figure 12**). Together with the knockdown experiments, this indicates that MT2A is not only a downstream effector of TGF-β1 but a positive regulator of the pro-fibrotic epithelial phenotype in this model.

**Figure 12:**
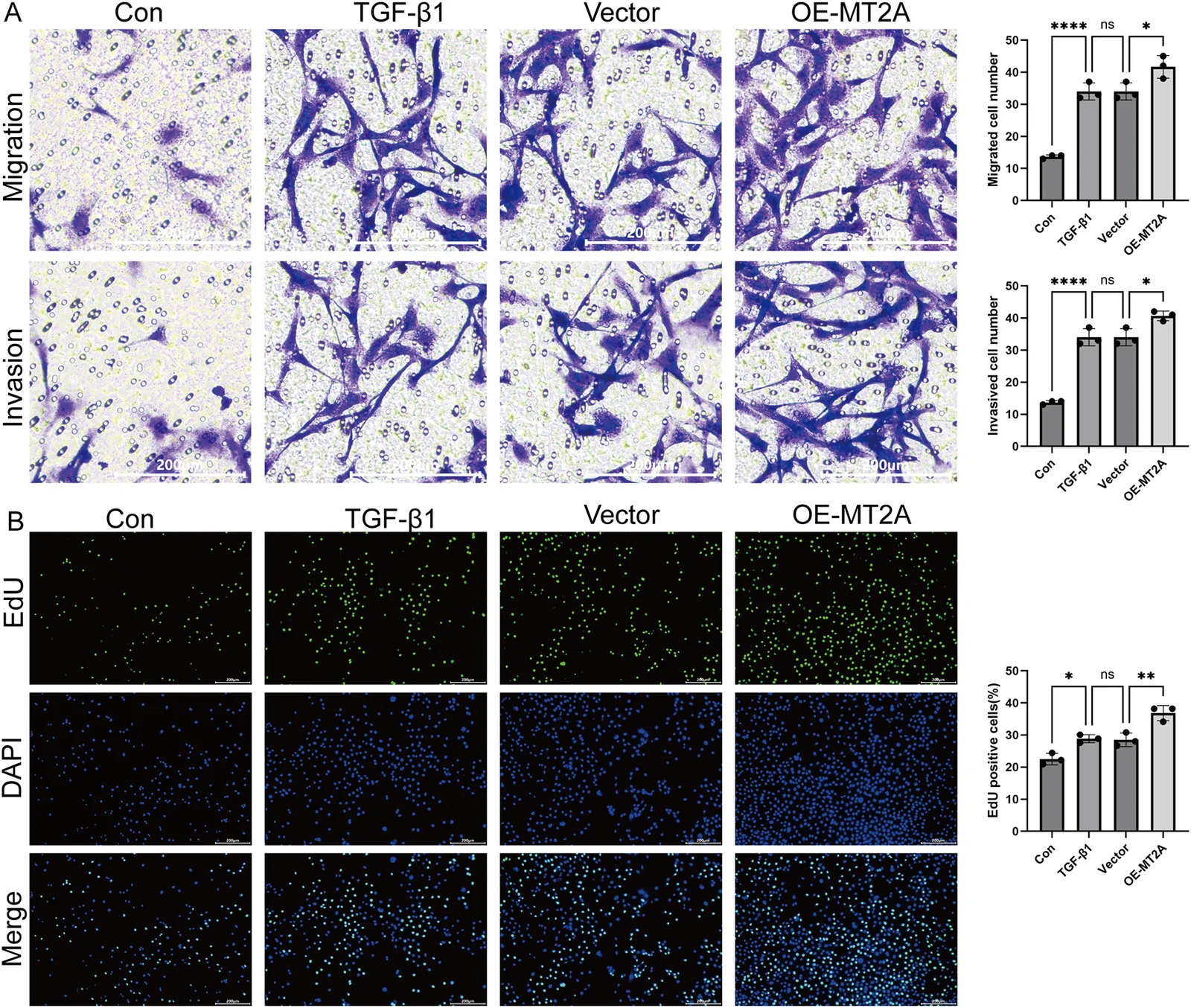
Overexpression of *MT2A* enhances TGF-β1-induced migration, invasion, and proliferation of BEAS-2B cells. **(A)**​ Transwell assays demonstrating that overexpression of *MT2A* (OE-*MT2A*) significantly enhances the migration and invasion of BEAS-2B cells induced by TGF-β1 stimulation. Scale bar, 100μm. **(B)**​ EdU proliferation assays showing that TGF-β1-induced cell proliferation is further promoted in BEAS-2B cells overexpressing MT2A. Scale bar, 50μm. Data are presented as mean ± SD (n = 3); *P < 0.05, **P < 0.01, ***P < 0.001, ****P < 0.0001.

To clarify the molecular-level regulatory role of MT2A in the pro-fibrotic process, we further conducted overexpression experiments in BEAS-2B cells. As shown in **Figure 13**, TGF-β1 stimulation significantly upregulated the mRNA and protein expression of MT2A, S100A8, COL1A1, VIM, FN1, and downregulated *CDH1*/E-cad expression, further confirming its ability to induce EMT. Based on this, overexpression of *MT2A* (OE-*MT2A*) further amplified the upregulation of TGF-β1 on the aforementioned S100A8 and EMT markers (COL1A1, VIM, FN1) and inhibited *CDH1*/E-cad expression. Mechanistically, overexpression of *MT2A* led to a significant increase in the mRNA and protein expression levels of its predicted downstream factor, S100A8 (**Figure 13**). Together with the loss-of-function data, these results indicate that MT2A promotes S100A8 expression and acts as an epithelium-associated upstream regulator of epithelial activation that sustains the EMT program through S100A8.

**Figure 13:**
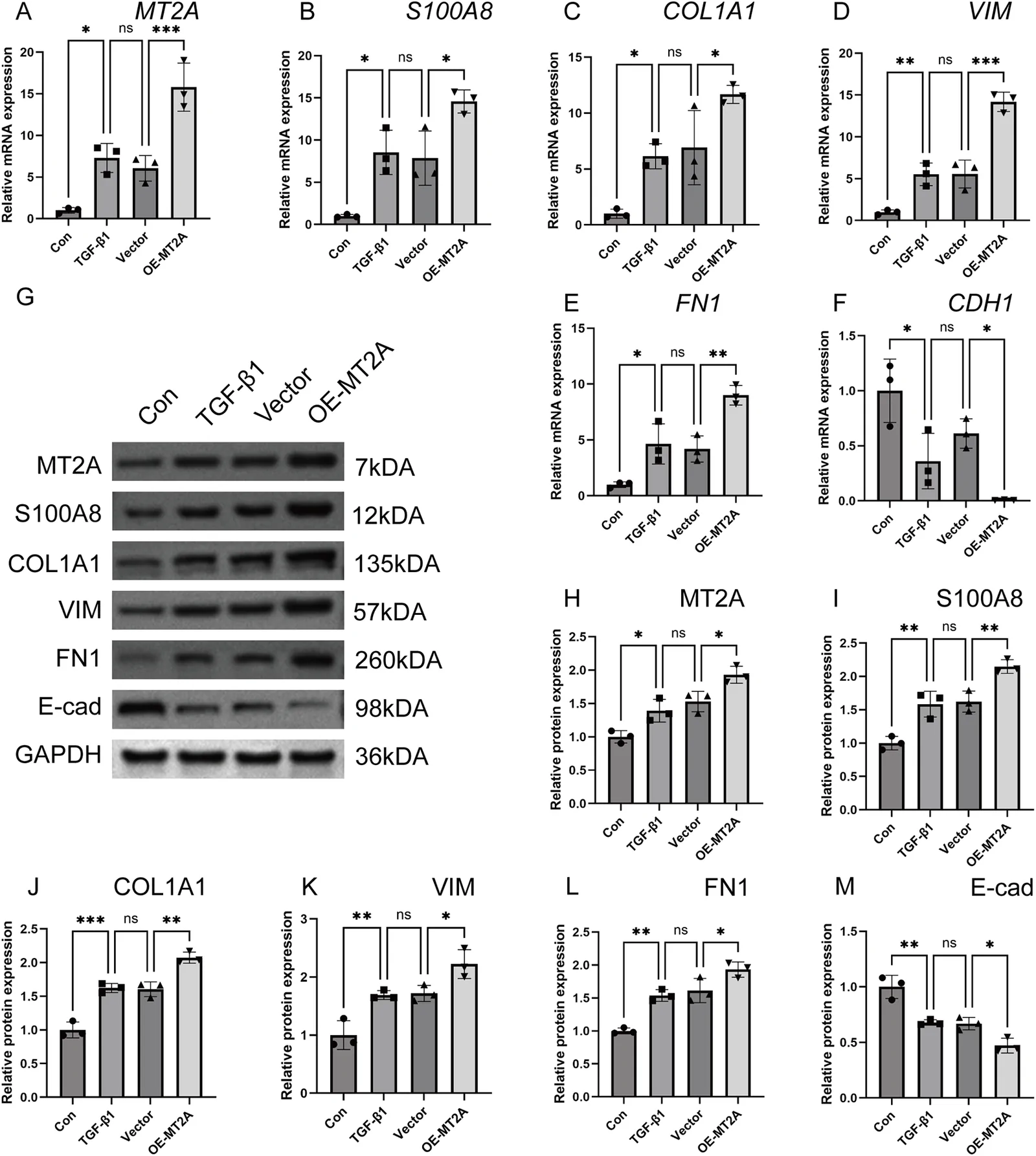
Overexpression of *MT2A* enhances TGF-β1-induced the expression of MT2A/S100A8 and EMT in BEAS-2B cells. **(A–F)**​ qRT-PCR analysis of the relative mRNA expression levels of *MT2A* **(A)**, *S100A8* **(B)**, *COL1A1* **(C)**, *VIM* **(D)**, *FN1* **(E)**, and *CDH1* **(F)** in BEAS-2B cells. **(G)**​ Western blotting images and quantitative analysis of protein levels for MT2A/S100A8 and EMT in BEAS-2B cells. **(H–M)**​ Western blotting analysis of the relative protein expression levels of MT2A **(H)**, S100A8 **(I)**, COL1A1 **(J)**, VIM **(K)**, FN1 **(L)**, and E-cad **(M)**. Data are presented as mean ± SD (n = 3); *P < 0.05, **P < 0.01, ***P < 0.001, ****P < 0.0001.

### 3.14 S100A8 is required for MT2A-driven EMT in BEAS-2B cells

Sufficiency does not by itself establish necessity. We therefore performed the reciprocal epistasis experiment, silencing *S100A8* in *MT2A*-overexpressing, TGF-β1-stimulated BEAS-2B cells. *S100A8* knockdown left MT2A unaltered at both the mRNA and protein level in vector- and OE-*MT2A*-transfected cells (**Figures 14A, G, H**), confirming that the hierarchy is strictly unidirectional and that S100A8 exerts no feedback on its upstream regulator. Critically, loss of *S100A8* abolished the pro-fibrotic gain conferred by *MT2A* overexpression: COL1A1, VIM and FN1 were reduced to levels indistinguishable from vector+si*S100A8* controls at both the transcript and protein level, and *CDH1*/E-cad was restored to an equivalent extent (**Figures 14B–F, I–M**). For every marker examined, the OE-*MT2A*+si*S100A8* and Vector+si*S100A8* groups did not differ significantly, indicating that MT2A confers no pro-fibrotic advantage once *S100A8* is removed. Taken together with the converse rescue, these reciprocal perturbations satisfy both criteria of an epistatic relationship—S100A8 is sufficient to reinstate EMT when *MT2A* is depleted and necessary for EMT when *MT2A* is in excess—thereby establishing S100A8 as the obligate downstream effector of MT2A and defining a linear, directional MT2A→S100A8→EMT axis in bronchial epithelial cells.

**Figure 14:**
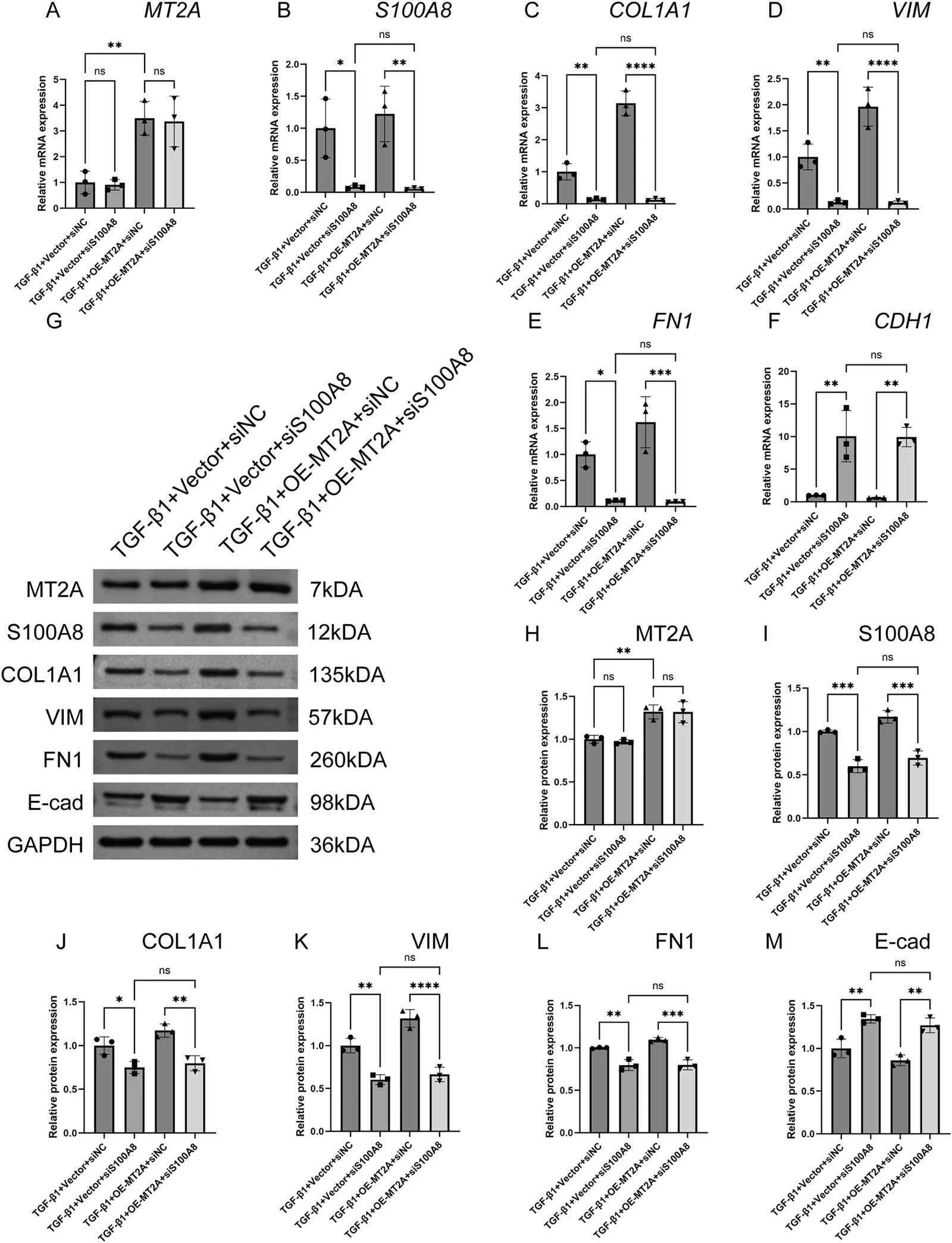
*S100A8* is required for MT2A-driven EMT in BEAS-2B cells. Cells were co-transfected with an OE-MT2A or empty vector together with siS100A8 or siNC, and stimulated with TGF-β1 (15 ng/ml) for 48 h. **(A–F)** qRT-PCR analysis of the relative mRNA levels of *MT2A* **(A)**, *S100A8* **(B)**, *COL1A1* **(C)**, *VIM* **(D)**, *FN1* **(E)** and *CDH1* **(F)**, normalized to GAPDH. **(G)** Representative immunoblots of MT2A, S100A8, COL1A1, VIM, FN1 and E-cad, with GAPDH as loading control. **(H–M)** Densitometric quantification of MT2A **(H)**, S100A8 **(I)**, COL1A1 **(J)**, VIM **(K)**, FN1 **(L)** and E-cad **(M)**, normalized to GAPDH and expressed relative to the TGF-β1+Vector+siNC group. Data are presented as mean ± SD (n = 3); *P < 0.05, **P < 0.01, ***P < 0.001, ****P < 0.0001.

## 4 Discussion

In this study we combined interpretable machine learning, single-cell VGKO, structural simulation and multi-level experimental validation to define an MT2A-S100A8 axis that drives EMT in PF. Beginning from an unbiased survey of five transcriptomic cohorts, our pipeline progressively narrowed a 55-gene fibrotic signature to a single, previously uncharacterized candidate driver, MT2A, and then nominated S100A8 as its principal downstream effector. The convergence of orthogonal computational and wet-laboratory evidence on the same molecular relationship is the central strength of the work and the basis for the mechanistic model we propose.

A key methodological contribution is the use of explainable artificial intelligence to move beyond association. Whereas conventional differential-expression analysis ranks genes by statistical separation alone, SHAP analysis of a high-performing LASSO classifier quantified both the magnitude and the direction of each gene’s contribution to the fibrotic phenotype, identifying MT2A as a strongly and positively weighted predictor. Single-cell RNA-seq showed that *MT2A* was robustly expressed across major circulating immune lineages; accordingly, VGKO of *MT2A* singled out *S100A8* as the most strongly perturbed transcript, with fewer than 2% of profiled genes significantly altered. This predicted regulatory dependency was corroborated by the concordant upregulation of both genes in bulk transcriptomic data and, decisively, by loss- and gain-of-function experiments showing that *MT2A* sustains *S100A8* at the mRNA level, thereby establishing *MT2A* as an upstream (transcriptional/post-transcriptional) regulator of S100A8 and fixing the direction of the axis.

The downstream effector our pipeline nominated is itself well supported by independent clinical and functional data. S100A8, together with its dimerization partner S100A9, is consistently elevated in the serum and bronchoalveolar lavage fluid of patients with IPF, and elevated levels are associated with shorter progression-free and overall survival (42) and with poor prognosis during acute exacerbation (43). Importantly, S100A8/A9 is not merely a passive marker: acting largely as a secreted alarmin through the receptor for advanced glycation end-products (RAGE), it promotes fibroblast proliferation and myofibroblast differentiation, and neutralizing antibodies against S100A8/A9 attenuate BLM-induced PF in mice (44). Du et al. demonstrated that an S100A8-high neutrophil subpopulation drives coal-dust-induced PF through TLR4-mediated communication with macrophages, and that the S100A8 inhibitor paquinimod disrupts this axis and attenuates fibrosis (45). Tan et al. revealed that loss of CEBPA in alveolar epithelial cells drives PF in part through S100A8/A9 upregulation, and that S100A8/A9 inhibition attenuates experimental lung fibrosis, positioning S100A8/A9 as a key downstream effector of impaired epithelial identity (46). Two independent studies have confirmed that S100A8 is highly expressed in the fibrotic lung, arising from profibrotic neutrophils and CEBPA-deficient alveolar epithelial cells, collectively establishing S100A8 upregulation as a convergent feature in PF. These studies predominantly describe S100A8/A9 as an extracellular, myeloid-derived alarmin; the MT2A-S100A8 axis defined here instead concerns intracellular S100A8 within epithelial cells. Our principal advance is therefore not the demonstration that S100A8 is profibrotic per se, but the identification of MT2A as an upstream regulator of S100A8 within epithelial cells, linking an epithelial stress response to an established innate-immune amplification pathway.

MT2A is a candidate upstream regulator of the NF-κB-responsive alarmin S100A8 within the injured epithelium. MT2A is a small, cysteine-rich, metal-binding protein whose canonical roles lie in zinc homeostasis, redox buffering and cytoprotection (10, 11, 47). These same activities place it plausibly upstream of S100A8: metallothioneins act as zinc chaperones and electrophilic scavengers and have been identified as intracellular modulators of NF-κB signaling through the sequestration and controlled release of zinc (48, 49), and the MT-zinc axis is increasingly recognized as a regulator of immune and inflammatory programs (50). Because S100A8 is an NF-κB-responsive alarmin, MT2A is well positioned to influence its transcription, and we therefore hypothesized that MT2A acts upstream of S100A8 within the injured epithelium. What has remained unknown is how S100A8 is controlled in this compartment. Our data identify MT2A as one such epithelium-associated regulator that sets S100A8 levels and thereby feeds into the fibrotic program.

We next asked how MT2A controls S100A8 and whether the two proteins also interact, using functional perturbation, biochemical assays and in silico modeling. Functionally, in TGF-β1-stimulated bronchial epithelial cells, loss- and gain-of-function manipulation of MT2A produced reciprocal effects: knockdown suppressed migration, invasion and proliferation, lowered S100A8 at both the mRNA and protein levels, and reversed the EMT signature (restoring *CDH1*/E-cad while lowering COL1A1, VIM and FN1), whereas overexpression amplified each response. Because these changes were evident at the transcript level, they indicate that MT2A acts predominantly as an upstream regulator of S100A8 expression, most plausibly through the metallothionein–zinc–NF-κB axis outlined above. Consistent with a regulatory role *in vivo*, MT2A and S100A8 were co-upregulated in BLM-induced fibrotic lung alongside a coordinated surge of pro-inflammatory and chemotactic mediators (TNF-α, IL-6, IL-1β, CXCL1, CCL3), in keeping with the innate-immune and chemotactic pathways enriched in our analyses and with the recognized role of S100A8/A9 in neutrophil recruitment and alarmin signaling (12–14). Epistasis provides genetic evidence for this hierarchy. Re-expressing *S100A8* in *MT2A*-depleted cells restored S100A8 to control levels and fully reinstated the EMT program, so *S100A8* is sufficient to bypass the requirement for *MT2A*. Conversely, silencing S100A8 in *MT2A*-overexpressing cells removed every pro-fibrotic gain conferred by *MT2A*, so *S100A8* is also necessary. Together the two perturbations satisfy a genetic definition of epistasis and identify S100A8 as the obligate downstream effector through which *MT2A* licenses EMT.

Our structural and biochemical data show that MT2A and S100A8 also associate at the protein level. Protein-protein docking followed by all-atom MD indicated a stable MT2A-S100A8 complex, supported by convergent RMSD and radius-of-gyration profiles, persistent interfacial hydrogen bonding and a favorable MM-PBSA energy with identifiable hotspot residues. Co-IP recovered the two proteins from a common complex in cells, and SPR provided independent evidence of direct binding, with an apparent dissociation constant of 88 ± 20 nM. Their shared cytoplasmic localization in TGF-β1-stimulated epithelial cells provides a spatial context compatible with such an interaction. We interpret this engagement as complementary to the expression changes we observed, because a direct protein-protein interaction would not by itself account for the S100A8 mRNA changes. Whether the complex contributes to S100A8 stabilization, localization or downstream function, and how it relates to the transcriptional control described above, remains to be defined. These observations also raise a translational possibility. An AI-guided virtual screen nominated BRD-K54403101 as a computationally predicted MT2A binder, indicating that the same pipeline that identifies a driver can also surface a chemical starting point. Because this nomination rests entirely on docking and simulation and has not been tested experimentally, BRD-K54403101 should be regarded only as a computational hypothesis for future validation.

Several limitations temper interpretation. First, our experiments distinguish transcriptional from post-transcriptional control only indirectly: the parallel loss of *S100A8* mRNA and protein is consistent with a requirement at the level of expression, but we cannot exclude a contribution from mRNA stability or translation. Second, the structural models and MM-PBSA estimates are predictions. They are constrained by MT2A’s small size and metal-dependent folding and are reported as relative; the measured affinity of the complex therefore rests on the SPR measurement, not on computation. Third, S100A8 was assayed here as an isolated protein, whereas in tissue it acts largely as an S100A8/A9 heterodimer, which we did not reconstitute. Fourth, our findings derive from an immortalized bronchial line with TGF-β1-induced EMT and from a single BLM model, neither of which reproduces the cellular heterogeneity of human PF; in particular, we did not validate the axis in A549 cells, primary alveolar epithelial cells, lung organoids or patient-derived epithelial cells. In addition, epithelial co-expression of MT2A and S100A8 in lung tissue was inferred from immunofluorescence morphology without concurrent epithelial lineage markers; confirmation will require multiplex staining with markers such as *EPCAM* or *SFTPC*. Fifth, we have not defined the molecular route by which MT2A raises S100A8. Our data show that MT2A is required for S100A8 mRNA and protein abundance and that the two proteins also interact, but we did not test S100A8 promoter activity, mRNA stability, p65/NF-κB activation or zinc-dependent regulation; the metallothionein-zinc-NF-κB model therefore remains a hypothesis. Establishing therapeutic potential will require *in vivo MT2A* perturbation, patient-derived epithelial systems and direct testing of the candidate compound.

## 5 Conclusion

Combining interpretable machine learning, single-cell VGKO and epithelial perturbation identified MT2A as an upstream regulator of S100A8 in TGF-β1-induced EMT. MT2A and S100A8 co-exist in a common complex in cells and bind directly *in vitro* (apparent KD = 88 nM by SPR), and reciprocal epistasis places S100A8 downstream of MT2A as the effector through which MT2A sustains EMT: removing S100A8 eliminated the pro-fibrotic effect of MT2A overexpression. The axis is therefore genetically defined and experimentally tractable. BRD-K54403101 emerged from AI-guided screening as a computational starting point for pharmacological testing, which has not yet been performed.

## Data Availability

The raw data supporting the conclusions of this article will be made available by the authors, without undue reservation.

## Supplementary material

Supplementary Figure 1 Single-cell RNA-seq analysis in PF and expression of key genes. **(A, B)** Sequencing depth versus detected genes and mitochondrial reads; cells are colored by sample. **(C)** Highly variable genes (red), with the top ten labeled. **(D)** t-SNE projection colored by unsupervised clusters (0–23). **(E)** Dot plot of canonical markers for ten cell populations; dot size and color denote expressing-cell percentage and scaled mean expression. **(F)** t-SNE colored by cell type and split into control and PF. **(G–J)** Feature plots of log-normalized *MT2A, CRIP1, SLCO4A1* and *IL18R1. PBMC*, peripheral blood mononuclear cell.

Supplementary Figure 2 AI-Driven Drug Screening and Analysis with DrugReflector. **(A)** The volcano plot highlights the top 10 drugs most relevant to the disease, BRD-K54403101 is ranked first. X-axis representing the logit score and the Y-axis representing probability. **(B)** 3D structure of BRD-K54403101. **(C)** Structural of the MT2A-BRD-K54403101 complex. **(D)** Analysis of the MD stability of the MT2A-BRD-K54403101 complex, including RMSD, RMSF, Rg, hydrogen bonds, SASA, and Gibbs FEL. **(E)** MM-PBSA results of the MT2A protein-BRD-K54403101 complex, statistical analysis of residue contribution breakdown and binding free energy components.
