# Supplementary material for: Metallothionein-2A licenses an S100A8 alarmin program to drive epithelial-mesenchymal transition in pulmonary fibrosis
Source: Front Immunol. 2026 Oct 22;17:1920411. doi: 10.3389/fimmu.2026.1920411 (PMC13735613; doi:10.3389/fimmu.2026.1920411)
Supplement: Supplementary file 1 [file DataSheet1.pdf]

山东省立医院动物实验伦理审查表

Animal Experimental Ethical Inspection Form of Shandong Provincial Hospital

编号（No）：No.2024-038

<table>
<tr><td rowspan="14">申请人填写的相关信息 (Related information Filled by Applicant)</td><td colspan="2">申请单位
Name of Organization</td><td colspan="3">山东第一医科大学
附属省立医院 呼吸与危重症医学科</td><td>经费代码
Funding Code</td><td>2024FY039</td></tr>
<tr><td colspan="2">申请人
Applicant</td><td colspan="2">范 锐</td><td colspan="2">申请人学历
Education of Applicant</td><td>博 士</td></tr>
<tr><td colspan="2">技术职称
Professional Title</td><td colspan="2">主治医师</td><td colspan="2">操作培训证号
Operator Training Certificate ID Number</td><td></td></tr>
<tr><td colspan="2">实验名称
Experiment Title</td><td colspan="5">甲氨蝶呤诱导肺纤维化小鼠的关键基因研究</td></tr>
<tr><td colspan="2">质量合格证编号
Number of Quality Certification</td><td colspan="2"></td><td colspan="2">动物实验设施合格证编号
Number of Experimental Facilities Certification</td><td></td></tr>
<tr><td rowspan="4">拟进动物情况</td><td>动物来源
Source of Animal</td><td colspan="5">北京维通利华实验动物技术有限公司</td></tr>
<tr><td>品种品系
Species of Strain</td><td>C57BL/6J</td><td>等级
Grade</td><td>SPF</td><td>规格
Specifications</td><td>6 周龄</td></tr>
<tr><td>数量
Number</td><td colspan="2">♂ 12 只、♀ 0 只，
共 12 只</td><td colspan="2">申请日期
Application date</td><td>2026 年 04 月 13 日</td></tr>
<tr><td>进驻日期
Entering Date</td><td colspan="2">2026 年 05 月 14 日</td><td colspan="2">结束日期
Ending date</td><td>2026 年 06 月 20 日</td></tr>
<tr><td colspan="7">实验要点，包括实验目的、实验方法、观测指标、实验结束后处死动物的方法等(Aim of Experiment, Outline of Experiments, Experimental Methods, Observational Index, Executing Animal Method, et al)</td></tr>
<tr><td colspan="7">一、实验目的：本研究旨在通过建立博来霉素诱导的小鼠肺纤维化模型，利用高通量测序等技术筛选并验证在此病理过程中发挥关键作用的差异表达基因。本实验的核心目的是在分子层面阐明博来霉素肺毒性的潜在机制，为临床早期预警药物性肺损伤、评估个体化用药风险提供潜在的生物标志物，并为开发针对性的干预策略提供新的治疗靶点。
二、实验方法：本研究使用 12 只 SPF 级 C57BL/6J 小鼠（雄性，6 周龄），适应性饲养一周后，采用随机数字表法将其均分为两组：对照组与模型组，每组 6 只。
模型组：麻醉后气管内一次性滴注博来霉素溶液，给药剂量为 3 mg/kg，溶于生理盐水。
对照组：在相同时间点，腹腔注射等体积的生理盐水。
三、观测指标
1. 每日观察小鼠精神状态、活动度、呼吸状况，每周称重 1 次。
2. 肺纤维化程度：肺组织病理学检查（HE 染色观察炎症，Masson 染色观察胶原纤维）
3. 分子机制指标：CA4 mRNA 和蛋白表达水平。
EMT 相关标志物：E-CAD、VIM、COL1 和 α-SMA 的 mRNA 和蛋白表达水平。
四、实验结束后处死动物的方法：颈椎脱臼法处死</td></tr>
<tr><td colspan="2" rowspan="2">申请人签名
Signature of Applicant</td><td rowspan="2">范锐</td><td colspan="2" rowspan="2">联系电话
Telephone</td><td>办公室(O)</td><td>0531-68779559</td></tr>
<tr><td>移 动(M)</td><td>13553175636</td></tr>
</table>

<table>
<tr><td>申请者声明
Announcement of Applicant</td><td colspan="4">我将自觉遵守实验动物福利伦理原则，随时接受实验动物伦理委员会的监督与检查，如违反规定，自愿接受处罚。(I will abide by the rules of the Animal Experimental Ethics Committee, accept the supervision and inspection of the Animal Experimental Ethics Committee, and accept the punishment if any infringement.)

声明人签名(Signature):
2026 年 4 月 13 日</td></tr>
<tr><td>审查依据
Inspection Contents</td><td colspan="4">1. 该项目是否必须用实验动物进行实验，即能否用计算机模拟、细胞培养等非生命方法替代动物或用低等动物替代高等动物进行实验(Does laboratory animal must be used in the project? Could other methods such as computer simulation,cell culture or using the low-grade animal instead of the high – grade animal?)
2. 表中所填申请人资格和所用动物的品种品系、质量等级、规格是否合适，能否通过改良设计方案或用高质量的动物来减少所用动物的数量(Are the qualification of applicant, species or strain, grade and specifications of animals suitable? Could the quantity of animals be reduced by improving the study design or using high quality animals?)
3. 能否通过改进实验方法、调整实验观测指标、改良处死动物的方法，来优化实验方案、善待动物(Could the study design and animal treatment be refined by ameliorating experimental method, adjusting observational index, executing animal method?)</td></tr>
<tr><td rowspan="3">审查结果
Results of Inspection</td><td>课题负责人意见
Project Director Attitude</td><td>同意
Agree 口</td><td>不同意
Disagree 口</td><td>签名
Signature</td></tr>
<tr><td>实验动物管理委员会意见
Laboratory Animal Managment Committee</td><td>同意
Agree 口</td><td>不同意
Disagree 口</td><td>签名
Signature</td></tr>
<tr><td>实验动物伦理委员会意见
Attitude of the Animal Care & Welfare Committee</td><td>同意
Agree 口</td><td>不同意
Disagree 口</td><td>签名
Signature</td></tr>
<tr><td colspan="4">备注(Remark)</td><td>签章(Stamp)
2026年 5 月 7 日</td></tr>
</table>

说明：

1. 编号由实验动物伦理委员会分配并填写。
2. 表格所有填写内容请用签字笔填写或电脑打印(签名处除外)。项目负责人、执行人及合作单位负责人均需在声明人签字栏签字。
3. 需随本表递交相关审查资料如实验方案、课题标书等。要求写明项目的意义、必要性、项目中有关实验动物的用途、饲养管理或实验处置方法、预期出现的对动物的伤害、处死动物的方法、项目进行中涉及动物福利和伦理问题的详细描述。
