# Supplementary material for: Metallothionein-2A licenses an S100A8 alarmin program to drive epithelial-mesenchymal transition in pulmonary fibrosis
Source: Front Immunol. 2026 Oct 22;17:1920411. doi: 10.3389/fimmu.2026.1920411 (PMC13735613; doi:10.3389/fimmu.2026.1920411)
Supplement: Supplementary file 2 [file DataSheet2.pdf]

frontiers

The International Society for the Advancement of Cytometry (ISAC) has highlighted the importance of including comprehensive methodological information to ensure data reproducibility and reliability. In line with this, Frontiers in Immunology now requires authors to submit a checklist for manuscripts that involve flow or mass cytometry. This checklist helps standardize the reporting process, improving the quality and transparency of published data. By doing so, we support scientific progress, making it easier for other researchers to replicate and validate experiments.

This form should be submitted with any manuscripts using flow or mass cytometry.

Sample/specimen/material description

☐ Total blood

☐ PBMCs

☐ Organ digests

Other Human bronchial epithelial cells (BEAS-2B line)

Did the samples suffer any treatment before or after incubation with the antibodies?

☑ Drug TGF-β1 (0-30 ng/ml)

☐ Cell permeabilization ______________________

☑ Dye Annexin V-FITC

☑ Propidium iodine

☐ Not applicable

Other ______________________________________

Instrument and antibodies

Name of the Cytometer BD FACSCanto II (BD Biosciences)

**Antibodies and targets**	**Fluorochrome/ Metal**	**Catalog number/Company**
e.g. anti-CD4	FITC	Cat. XXX/ XXX ltd.
Annexin V	FITC	BMS500FI-300 Invitrogen

Propidium iodide	PI	BMS500FI-300,Invitrogen

frontiers

Data analyses

1. Name of the software FlowJo (v10.8, BD Biosciences)
2. Reference gating strategy in the manuscript or supplementary material

Gating strategy in (eg Figure X) Figure 10B
